# Motile Living Biobots Self‐Construct from Adult Human Somatic Progenitor Seed Cells

**DOI:** 10.1002/advs.202303575

**Published:** 2023-11-30

**Authors:** Gizem Gumuskaya, Pranjal Srivastava, Ben G. Cooper, Hannah Lesser, Ben Semegran, Simon Garnier, Michael Levin

**Affiliations:** ^1^ Allen Discovery Center at Tufts University and Department of Biology Tufts University Medford MA 02155 USA; ^2^ Wyss Institute for Biologically Inspired Engineering Harvard University Boston MA 02115 USA; ^3^ Federated Department of Biological Sciences New Jersey Institute of Technology Newark NJ 07102 USA

**Keywords:** biobot, bioengineering, emergence, morphogenesis, repair, self‐assembly

## Abstract

Fundamental knowledge gaps exist about the plasticity of cells from adult soma and the potential diversity of body shape and behavior in living constructs derived from genetically wild‐type cells. Here anthrobots are introduced, a spheroid‐shaped multicellular biological robot (biobot) platform with diameters ranging from 30 to 500 microns and cilia‐powered locomotive abilities. Each Anthrobot begins as a single cell, derived from the adult human lung, and self‐constructs into a multicellular motile biobot after being cultured in extra cellular matrix for 2 weeks and transferred into a minimally viscous habitat. Anthrobots exhibit diverse behaviors with motility patterns ranging from tight loops to straight lines and speeds ranging from 5–50 microns s^−1^. The anatomical investigations reveal that this behavioral diversity is significantly correlated with their morphological diversity. Anthrobots can assume morphologies with fully polarized or wholly ciliated bodies and spherical or ellipsoidal shapes, each related to a distinct movement type. Anthrobots are found to be capable of traversing, and inducing rapid repair of scratches in, cultured human neural cell sheets in vitro. By controlling microenvironmental cues in bulk, novel structures, with new and unexpected behavior and biomedically‐relevant capabilities, can be discovered in morphogenetic processes without direct genetic editing or manual sculpting.

## Graphical abstract

Lung epithelial cells from human donors assemble into a self‐propelled biobot. Confronted with a damaged sheet of human neural cells, these Anthrobots induce repair across the injury. This novel biorobotics platform reveals morphological and behavioral plasticity of human cells, without genomic editing, and offers a roadmap for living meso‐scale robots made from patients’ own cells with many potential uses.

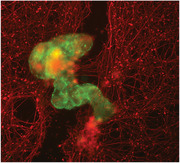

## 1 Introduction

What is the latent space of possible functional morphologies that cells, with a wild‐type genome, can be coaxed to construct?^[^ ^1^ ^]^ This question drives at the heart of fundamental issues in evolutionary, developmental, cell, and synthetic biology, and has been taken up by a rapidly growing field focusing on building new kinds of active living structures: biobots.^[^ ^2^ ^]^ This emerging multidisciplinary effort to control the behavior of cellular collectives has garnered much excitement for two main reasons. First, it offers the possibility of using engineering to reach outcomes that are too complex to micromanage directly, and hence promises to revolutionize efforts to produce complex tissues for clinical applications in regenerative medicine and beyond. Second, increased control over the morphology and behavior of cellular collectives by leveraging morphogenetic tissue plasticity could enable the development of self‐constructing living structures by design with predictable and programmable functional properties and numerous practical uses, greatly extending the current abilities of traditional fabrication practices in diverse fields as robotics,^[^ ^3^ ^]^ architecture, sustainable construction, and even space exploration.

In the last decade, interest in developing biological structures *de novo* has seen a rapid surge.^[^ ^4^ ^]^ Among these efforts, a subset of functional biogenic assemblies gave rise to a special class of motile synthetic structures dubbed *biobots*. Early examples of biobots are hybrids between biological cells and inert chemical substances supporting them, such as gels or 3D‐printed scaffolds.^[^ ^5^ ^]^ These assemblies incorporated living cells ranging from bacteria to diverse mammalian tissues such as nerve, muscle, and neuromuscular junctions (NMJs), as well as engineered cell lines with programmable features, all carefully crafted into diverse 3D scaffolds designed to harness and amplify the innate functionality of biological cells.^[^ ^6^ ^]^

A different approach resulted in Xenobots, the first fully‐biological biobots created by sculpting or molding amphibian embryonic cells into multicellular structures that can spontaneously locomote without external pacing.^[^ ^7^ ^]^ But it was not known how general these phenomena are, whether this kind of plasticity extended to mammals, or what the throughput of this technology can be. Thus, we sought to address whether the capacity of genetically unaltered cells to generate a self‐propelled, multicellular living structure in this way is unique to amphibian embryonic cells, and whether such a living structure can be built without needing to be individually sculpted or molded, but instead coaxed to self‐construct from an initial seed cell, resulting in a high‐throughput process wherein large numbers of biobots can be grown in parallel.

Here, we introduce novel, multicellular, fully biological, self‐constructing, motile living structures created out of human lung epithelium. We refer to them as Anthrobots, in light of their human origin and potential as a biorobotics platform.^[^ ^2^, ^8^ ^]^ We quantified their emergent natural, baseline properties as an essential background characterization of their native capacities which will serve as targets for future efforts to reprogram form and function for useful purposes. Anthrobots self‐construct in vitro, via a fully scalable method that requires no external form‐giving machinery, manual sculpting, or embryonic tissues and produces swarms of biobots in parallel. They move via cilia‐driven propulsion,^[^ ^9^ ^]^ living for 45–60 days. We quantitatively characterized the range of movement and morphological types, showing that their behaviors are strongly correlated with specific features of their anatomy. The ability of adult, somatic, human cells to form a novel functional anatomy, with unique behaviors, reveals that this plasticity is not restricted to amphibian or embryonic cell properties, and is a fundamental feature of wild‐type cells that requires no direct genetic manipulation to unlock. Furthermore, we found that Anthrobots exhibit a highly surprising behavior given their origin as static airway epithelium: they can move across scratches in (human) neuronal monolayers and induce gap closures across these scratches. Numerous in vitro and in vivo uses of such functional living structures can be envisioned, especially because they can now be made from the patient's own cells.^[^ ^10^ ^]^

We developed the Anthrobots by leveraging normal human bronchial epithelial (NHBE) cells’ native tissue plasticity and unlocked a novel morphology, which is not apparent from the reliable, default developmental patterning of airway epithelium, in order to fulfil target functional and structural needs of creating a self‐constructing, multi‐cellular, 3D, motile living structure of human‐origin. Airway organoids with apical out tissue organization, yielding ciliated spheroids anatomically similar to the Anthrobots, have very recently been shown using different protocols,^[^ ^11^ ^]^ each starting from individual normal bronchial epithelial cells isolated from airway epithelium extracts. Apical out spheroids made out of intact airway epithelium extracts have also been produced^[^ ^12^ ^]^). Each one of these three protocols is optimized for different priorities such as ease of organoid access during its development,^[^ ^11c^ ^]^ structural uniformity in final products,^[^ ^11a^ ^]^ and ability to easily modulate resulting organoid size.^[^ ^11b^ ^]^ The common denominator between these parallel advances is that they are characterized as organotypic cultures exclusively, enabling scientists to investigate lung anatomy, function, and pathology. Beyond native tissue recapitulation, these constructs’ abilities as functional assemblies, range of behavioral and morphological patterns, as well as functional correlations between these patterns have yet to be explored.

These three methods, plus the one detailed in this paper, constitute convergent but distinct technical approaches toward producing the novel morphology of cilia‐out spheroids derived from human airway epithelium. These four protocols for creating cilia‐covered NHBE‐derived spheroids differ in their apical orientation from the earlier established approaches for creating traditional airway organoids where cilia develop as lining the lumen.^[^ ^13^ ^]^ Boecking & Walentek^[^ ^11c^ ^]^ grow airway organoids as embedded in a collagen‐rich matrix (as opposed to the traditional Matrigel approach) and also cultures them in air‐liquid‐interface (ALI) inserts, which have traditionally been used with NHBEs for 2D differentiation into airway epithelium, enabling ease of access to the airway organoids during their developmental course.^[^ ^11c^ ^]^ After this initial ALI culture period of 14 days, the mature airway organoids are dissolved from the collagen matrix and replated into a fresh same matrix of similar composition (to remove catabolites) for another 14 days. It is in this second 14 day period that cilia localization on the surface is accomplished by administering R‐Spondin‐2 (RSPO2) and Noggin into the matrix. Accordingly, this Boecking & Walentek method consists of two consecutive 2‐week periods of matrix‐embedded growth and differentiation: first period without and the second period with RSPO2&Noggin.

The method introduced in our paper is most similar to the Boecking & Walentek method in that the initial proliferation of individual NHBEs into spheroids with cilia‐lined lumen is accomplished by culturing them as embedded in a gel‐based matrix. However, in our method, upon dissolution of spheroids from matrix at the end of this 14 day period, the cilia‐in spheroids are not plated back into the matrix, and instead, the cilia localization into the spheroid cortex is achieved by culturing these spheroids in low‐adhesive environments. Accordingly, cilia localization is observed within one week, making our method a faster (20 days between single cell to cilia‐coated spheroid as opposed to 28), less laborious (single matrix dissolution, as opposed to two), and potentially higher‐throughput (since each time matrix is dissolved, a certain percentage of spheroids are lost with it, as is also reported by Boecking & Walentek). The remaining two cilia‐out protocols (Stroulios and Wijesekara) are in contrast not leveraging the self‐construction ability of NHBEs, and instead form spheroids by means of cell aggregation in U‐bottom wells without the presence of a matrix, which in turn provide them with higher regularity of spheroid size (given each spheroid self‐assembles with a similar number of constituent cells).

The Stroulios et al method aggregates single cells in low‐adhesive micro aggregation chambers first, then transfers them into regular wells for differentiation in matrix‐free liquid environment with bronchial epithelial differentiation medium, achieving uniformity in organoid morphology and size.^[^ ^11a^ ^]^ Similarly, the Wijesekara et al method also first aggregates individual cells into spheroids, and then either transforms them into a matrix environment for differentiation, or keeps them in the liquid environment with bronchial epithelial differentiation medium, both approaches yielding apical‐out spheroids with the ability to control the resulting ciliated spheroid size by modulating the initial aggregate size.^[^ ^11^, ^14^ ^]^ In summary, our method facilitates differentiation as embedded in extracellular matrix, enabling spheroids to self‐construct, a feature the other two methods Stroulios and Wijesekara lack; though in turn they achieve higher spheroid regularity and control over spheroid size. The major difference between our method and the Boecking & Walentek method is that upon extraction of apical‐in spheroids from Matrigel, we simply culture the spheroids in a non‐adhesive environment and achieve polarity reversal and motility within a few days. However, the Boecking & Walentek method replates the spheroids into the Matrigel for another two weeks, during which period they administer RSPO2&Noggin in order to facilitate polarity reversal, and at the end of this period they dissolve the Matrigel again to finally harvest the apical out spheroids. We believe our method provides a simple, rapid, scalable, and high‐throughput protocol that harnesses biological cells’ ability to build themselves into multicellular complex structures. Despite their differences in methods, goals, and characterization metrics, these four novel protocols taken together help explore the space of possible morphologies of NHBEs and unravel the morphogenetic potential of human airway epithelium; they are thus significant steps for mapping the morphogenetic plasticity landscape of non‐embryonic wild‐type cells.

## 2 Results

### 2.1 Human Bronchial Epithelial Cells Self‐Construct into Multicellular Motile Living Architectures

**Figure 1:**
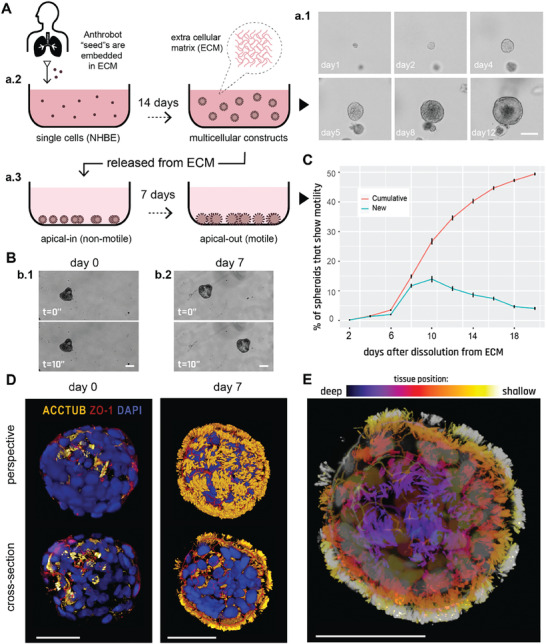
Human bronchial epithelial cells self‐construct into multicellular motile living architectures. A) Workflow for producing Anthrobots. NHBE cells’ apical‐in to apical‐out transition is facilitated by first culturing them in extra cellular matrix (ECM) under appropriate differentiation‐inducing conditions, during which time apical‐in spheroids self‐construct from single cells a.1), and upon the completion of this 14 day period a.2) by releasing mature spheroids from the ECM a.3) and continuing to culture them in low‐adhesive environment. B) Phase contrast images of an apical‐in b.1) and apical‐out b.2) spheroids, captured immediately after dissolution from ECM (day 0) and 7 days after dissolution (day 7), respectively. Day 0 spheroids show no motility, whereas day 7 spheroids show drastically increased motility. C) Percentage of cumulative (total fraction of motile spheroid since day 0) and newly motile spheroids (fraction of motile spheroid that reached motility since the previous time point) in the 3 weeks following dissolution. Out of the 2281 spheroids characterized total, ≈50% consistently showed no signs of motility (despite most having cilia) within this 3‐week period and are referred to as non‐movers. The data shown on this graph only include the motile bots, N = 1127. D) Immunostaining of two separate spheroids from day 0 and day 7 with a‐tubulin (cilia marker), Zonula occludens (ZO)‐1 (tight junction marker), and the nuclear stain 4',6‐diamidino‐2‐phenylindole (DAPI). Amount of multiciliate cells on the spheroid surface show a drastic increase by day 7. E) A day 7 Anthrobot with depth information to show full cilia coverage. Bots in panels D,E were immunostained with α‐tubulin (cilia marker), ZO‐1 (tight junction marker), and DAPI (nuclear stain). Colors represent tissue depth. All scalebars on this figure feature 50 um.

To explore the self‐organizing plasticity of morphogenesis without genomic change, we chose a cellular substrate in which such outcomes would be most surprising: adult, somatic, human airway tissues. To study and steer the in vitro morphogenesis of novel 3D tissues with motile appendages, we developed a novel protocol (**Figure** **1**A) that builds upon the existing ability of human bronchial epithelial progenitor cells to form multicellular spheroids (Figure 1a.1) with cilia‐lined lumina^[^ ^15^ ^]^ (i.e., apical‐in configuration). We modified this process by manipulating the culture environment such that it now yields cilia‐coated (i.e., apical‐out) spheroids, which exhibit spontaneous locomotive ability.

A key step in their construction, in order to obtain significant translocation, is the induction of cilia to face outward. Given that cilia naturally localize into the lumen due to the basal cells’ interaction with the surrounding high‐viscosity matrix, we hypothesized that changing the culture environment to a lower viscosity level (e.g., water‐based media instead of gel‐based matrix) may trigger the basal layer cells to migrate inward and allow the apical layer to take their place on the spheroid cortex.^[^ ^16^ ^]^ Thus, to trigger apicobasal polarity switching, we first grew airway organoids embedded in Matrigel as reported previously^[^ ^13^ ^]^ (Figure 1a.2), which does not yield either ciliated or motile spheroids. We have then proceeded to dissolve the surrounding matrix while keeping the spheroids intact and transferred them into a low‐adhesive environment (Figure 1a.3) and induced them with retinoic acid on a bidaily basis while performing media changes every 3 days. (Matrigel's elastic modulus is 450 Pa^[^ ^17^ ^]^ whereas water based liquid media's elastic modulus in this low‐adhesive environment is 2*10^9^ Pa). This new approach has enabled the originally apical‐in spheroids (that show no motility on day 0 as shown on Figure 1b.1) to became motile by day 7 (Figure 1b.2), featuring highly motile ciliary appendages on the spheroid surface. A high‐resolution high‐speed capture of ciliary movement in Anthrobots (supplemental video 1) show that they deploy a similar propelling strategy observed in multiciliate motile organisms.^[^ ^9^ ^]^

We next examined two aspects of the microenvironment as possible control parameters for properties of Anthrobot self‐assembly. First, we tested the role of matrix viscosity, which is known in other bioengineering contexts to impact diverse cell properties, from secretory profile^[^ ^18^ ^]^ to mechanical attributes.^[^ ^19^ ^]^ We observed that culture environments with higher viscosity levels than the protocol baseline result in decreased motility (Figure S2, Supporting Information) and size (Figure S3, Supporting Information), suggesting that low‐viscosity environments better facilitate the growth of functional bots as well as yield larger bots. Second, we examined cell seeding density as a factor for motility (Figure S4, Supporting Information) and size (Figure S5, Supporting Information). We set up three separate conditions: one with the default seeding density in Matrigel (x = 30 000 cells mL^−1^), one with double this density (2x = 60 000 cells mL^−1^), and one with half this default density (x/2 = 15 000 cells mL^−1^). After growing the bots under these three conditions for two weeks, while keeping all other protocol aspects constant, we dissolved the mature spheroids from the matrices and measured their size immediately. We then continued culturing the bots per usual maintenance protocol and measured their motility in a binary fashion (i.e., displacing mover or not) during the period where bots show peak motility (between days 9–20) on a bidaily basis. In both experiments, we observed significant differences in the resulting bot sizes and motility (on particular days) among different seeding density conditions, though the effect of introducing additional cells was not linear at any time point tested. These results show that the size and time course of maturation of motile bots could be modulated by altering the concentration of cells in the Anthrobot differentiation culture, suggesting initial cell seeding density to be a tractable control knob for Anthrobot morphology and function.

To characterize the temporal dynamics of motility initiation, we periodically (every other day) counted the number of motile spheroids for 3 weeks following dissolution and observed a sigmoidal motility profile with peak change in motility on day 10 (Figure 1C). We next confirmed that this drastic change in motility occurred as a result of a morphological reorganization event exposing cilia on the cortex (Figure 1D). We immunostained the spheroids on day 0 (pre‐motility) and day 7 (post‐motility) with DAPI and for the apical markers a‐tubulin (cilia marker) and ZO1 (tight junction marker), revealing a drastic increase in external multi‐ciliated cells on day 7 compared to day 0. Figure 1E shows the tissue organization within an ≈50‐micron depth of a typical Anthrobot. Upon observing increased multi‐ciliated cell presence in motile subjects, we sought to definitively attribute the emergence of motility to the presence of surface cilia. We administered the efficient blocker of cilia motion, ciliobrevin,^[^ ^20^ ^]^ and observed the expected drastic decrease in motility (See Figure S6, Supporting Information), confirming that the motility of Anthrobots is cilia‐driven.

### 2.2 Anthrobots Self‐Organize into Discrete Movement Types

**Figure 2:**
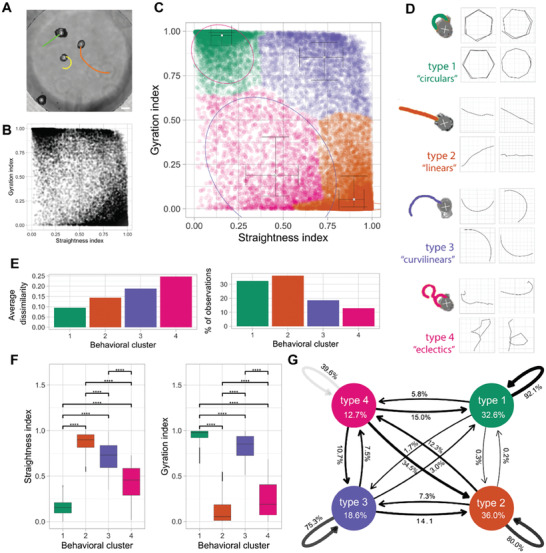
Anthrobots self‐organize into discrete movement types. A) Anthrobots display different movement types. Scalebar 100 uM. B) Distribution of all 30‐second periods in the analysis plotted by their straightness and gyration indices, showing signs of clustering near three of the 4 corners of the plot. C) Clustered scatter plot of all 30 s periods with centers of cluster marked and colored. D) Prototypical examples from each cluster with 30 s sample trajectories. E) Quantitative comparison of key characteristics of the four clusters in terms of intra‐cluster homogeneity “average dissimilarity”) and occurrence frequency (“% of observations”) which show that the largest clusters 1 and 2 have relatively low dissimilarity indicating these are the most consistent behavioral patterns. F) Comparison of gyration and straightness indices for each cluster with significance levels indicated, showing that each cluster occupies a unique, quantifiable position in the sample space. P‐value range after pairwise 2‐sample t‐test of 0 to 0.0001 corresponded to ****, 0.0001 to 0.001 corresponded to ***, 0.001 to 0.01 corresponded to **, 0.01 to 0.05 corresponded to * and 0.05 to 1 corresponded to ns. Cluster one had 6004 30 s periods, cluster two had 6700, cluster three had 3436 and cluster 4 had 2384. G) Markov chain showing state transitions between different clusters (same as in Figure 2F) and the degree of commitment to a given behavior (persistence), with the circular bots (type 1) as the most committed category with 92.1% chance of the next period being a circular if the current period is a circular. It is followed by linear and curvilinear, which are also relatively consistent at 80.0% and 75.3% respectively. Cluster 4, or the eclectics, as expected, are very unstable, with a consistency of only 39.6%. Cluster 4 seems to act as a sort of intermediate, since there is a substantial chance of the eclectics converting to linear (34.5%) or to a lesser degree circular (15.0%) or curvilinear (10.7%). The transition probability between circulars and linear and vice versa is the lowest and almost nonexistent, at 0.3% and 0.2% respectively. Linear, curvilinear, and circulars rarely convert into eclectics with a probability of 12.3%, 7.5%, 5.8% respectively (and when they do, it is most likely due to collisions or using eclectics as an intermediary).

Despite their wild‐type human genome and somatic origin, these self‐motile constructs exhibited a wide range of behaviors and an anatomy that differs from the species‐specific body morphology. To characterize this diverse landscape and uncover the developmental features of Anthrobots, next we sought to characterize these behavioral and morphological capabilities, and investigated a potential correlation between their form and function. One initial key task for Anthrobots, as with any new behavioral subject,^[^ ^21^ ^]^ is to determine whether its major morphological properties and activities are discrete, continuous, or uniform characters.^[^ ^22^ ^]^ Thus, we quantitatively analyzed their range of behavior modes in time‐lapse videos of ≈200 randomly‐selected motile spheroids (**Figure** **2**A, and Videos S2–S5, Supporting Information) for 5 h in groups of 4 or 5 Anthrobots, and extracted their movement trajectory coordinates. We then split up these 5 h‐long trajectories into 30 s periods to classify behavior with a higher degree of granularity and in an aggregate manner. To identify patterns within a potentially unlimited set of possible movements, we characterized these periods by how straight and/or circular they are as all possible trajectories can be explained together by these two properties. To this end, we used two main trajectory characterization metrics: straightness and gyration indices (see Experimental Section for detailed description of how these indices are calculated) and plotted all viable trajectorial periods along these two indices (Figure 2B). We then ran the unsupervised clustering algorithm Ward.D2, a common hierarchical clustering method (see methods for more details), on this 2D plot and observed four statistically distinct clusters to emerge (Figure 2C). Further investigation of these clusters reveals that each represents a distinct movement type: circular, linear, curvilinear and “eclectic” (Figure 2D). Further analysis of each cluster in terms of its homogeneity (measured by “average dissimilarity” index), which is a measure of the intra‐cluster variation, and its size (measured by “% of observations”) was performed (Figure 2E), as well as a quantitative comparison between different clusters along the two major movement indices (Figure 2F).

As a result of these behavioral characterization analyses, we observed that the circular bots (type 1, Figure 2D) score the highest on gyration and lowest on straightness indices (Figure 2F). They also have highly similar trajectories and are very common among the behaviors of Anthrobots given this cluster has the smallest homogeneity and a representation of over 30% of all the recorded periods (Figure 2E). We also observed that the linear bots (type2, Figure 2D) score the highest on straightness and lowest on gyration indices (Figure 2F). They have less homogeneity than circular bots but also have the greatest representation out of all clusters (Figure 2E). Accordingly, circular and linear bots together make up more than half of the population, and each have highly homogeneous populations. Finally, the third most common (Figure 2E) type of bot is the curvilinear bot (type 3, Figure 2D), which scores high on both the gyration and straightness indices (Figure 2F) and has the second most heterogeneous trajectories (Figure 2E). Bots with most disorganized trajectories and smallest representation in the overall population (Figure 2E) are the eclectic bots (type 4, Figure 2D), which score the lowest on both the gyration and straightness indices (Figure 2F) due to exhibiting eccentric trajectories that are often a combination of the other three types.

After having characterized each major movement type observed in Anthrobots, we next investigated the transition probabilities between each pair of behavior types. In order to estimate the stability of each trajectory and state transitions between different movement types, we used a Markov chain model shown on Figure 2G, which revealed the degree of commitment to a given behavior (persistence) and provided an ethogram of Anthrobot behavior. We observed that the most stable movement pattern for an Anthrobot is circular motion, followed by linear/curvilinear motion. The eclectics act more like an intermediate and over time, at least probabilistically, resolve into one of the 3 other categories. Therefore, we conclude that the vast majority of Anthrobot movements can be broken down into simpler, highly consistent patterns like linear, circular, curvilinear, with eclectics acting as a transient intermediary. The fact that Anthrobots exhibit movement types with high “consistency” and low rates of inter‐type conversion (e.g., between circulars and linear) suggests that Anthrobots self‐organize into discrete and stable movement types, each bot having a distinct motility fingerprint.

### 2.3 Anthrobots Self‐Organize into Distinct Morphological Types

**Figure 3:**
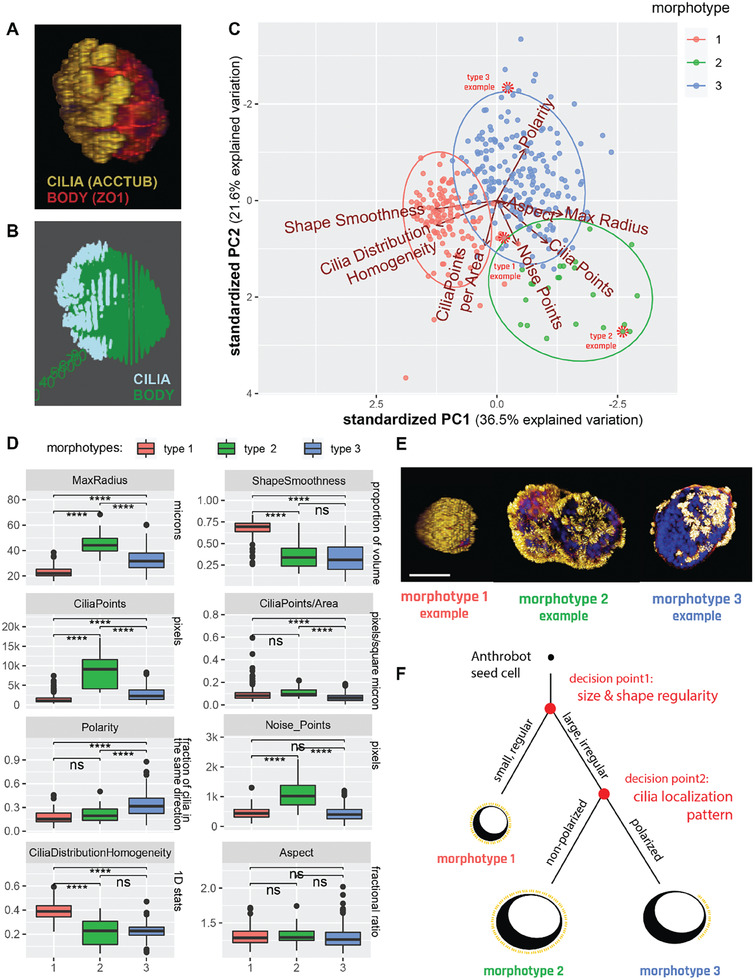
Anthrobots self‐organize into distinct morphological types. A) Anthrobot immunocytochemistry enables morphological classification pipeline. Sample immunological stain for cilia (acctub, i.e., acetylated α‐tubulin) and apical layer marked by the tight junction marker (ZO1) acquired as a complete 3D Z‐stack showing the Anthrobot body boundaries and cilia localization on the body. B) Binarized version of the sample immunological data, used as input to the morphological characterization pipeline. C) Binarized body and cilia information from 350 Anthrobots plotted along 8 morphological indices on an 8D cloud and clustered with the unsupervised Ward.D2 method, which identify global clusters based on the proximity of the centroids of locally emerging clusters and merging them together when applicable. PCA showing the three morphotypical clusters on the highest variation plane, marked by PC1 and PC2. Red dashed circles point to specific examples featured in panel E, selected from the cluster edges for distinct representation. D) Distinct morphotypes translate with significance to differences in real‐life morphological metrics, characterized by 8 variables from which the PCA was computed. P‐value range of 0 to 0.0001 corresponded to ****, 0.0001 to 0.001 corresponded to ***, 0.001 to 0.01 corresponded to **, 0.01 to 0.05 corresponded to * and 0.05 to 1 corresponded to ns. Cluster 1,2 and 3 in the analysis corresponded to the clusters in the PCA, with n = 125, 24 and 201 respectively. We ran a two‐sided, two‐sample t‐test on all pairs of clusters, for all 8 variables, which are then plotted here. E) Sample morphotype examples for Type 1, 2 and 3 chosen for their ability to best represent the cluster. Type 1 Anthrobots are small, regularly shaped, tightly and uniformly covered by cilia. Type 2 and 3 bots are larger, more irregularly shaped and have less tightly‐knit cilia patterns, with type 3 bots featuring significantly more polarized cilia coverage. Scalebar 50 uM. F) Decision tree of Anthrobot morphogenesis with two major checkpoints as revealed by the PCA hierarchy: first decision point is size/shape (has equal impact), second decision point is cilia localization pattern.

Having observed several distinct movement types, we next asked whether the range of Anthrobot morphologies was continuous or again composed of discrete categories.^[^ ^23^ ^]^ This question is important for both understanding the macro‐scale rules of self‐assembly, and for future efforts to control their functional properties. We hypothesized the primary parameters of this possible underlying morphological framework to be a function of the Anthrobots’ 3D shape and overall cilia distribution pattern, since Anthrobot motility is generated by cilia. Accordingly, we collected 3D structural data (**Figure** **3**a.1) from ≈350 Anthrobots through immunocytochemistry / immunofluorescence (ICC/IF) and confocal microscopy, focusing on shape and cilia distribution pattern properties, and binarized these morphological features (Figure 3a.2) to extract quantitative information on cilia and body boundaries. We then plotted this information for ≈350 Anthrobots along eight different morphological characterization indices we developed, each quantifying a different aspect of the Anthrobot shape and cilia pattern. (Figure 3B; Figure S7A, Supporting Information). The shape‐related indices among these eight formal morphological characterization indices included the ratio between the longest and shortest distance within a spheroid (i.e., “aspect”), longest distance within a spheroid (i.e., “max radius”), how invaginating or protruding the spheroid surface is (i.e., “shape smoothness”); the cilia‐related indices included the total area covered by cilia signal on a spheroid surface (“cilia points”), cilia signal per unit area on a spheroid surface (“cilia points/area”), proximity of current cilia point distribution to a complete random uniform distribution (“cilia distribution homogeneity”), how clustered the cilia are on a spheroid surface (“polarity”), and how many free‐floating cilia points there are that are not a part of a cluster (“noise points”). See Experimental Section for more details on how these morphological indices were calculated.

Next, we performed a Principle Component Analysis (PCA) followed by an unsupervised clustering algorithm on this 8D data cloud and observed the emergence of three statistically distinct clusters (Figure 3C), each representing a distinct morphological type (morphotype). Figure 3D shows a quantitative characterization of each cluster along 8 different morphological indices. This analysis revealed the following two characteristics to be the most important distinguishing factors (to an equal degree, both ranking the top place in Principle Component (PC)1 contributions) between different morphotypes: the size of the Anthrobot (measured by “max radius”), and the uniformity of its shape (measured by “shape smoothness”). These two most distinguishing characteristics formally describe type 1 bots to be significantly smaller in size and smoother (spherical) in its volume, while type 2 bots to be the largest and least uniformly shaped, and type 3 to be somewhere in between the two both. (See Materials and Methods section for PC1 and PC2 contribution rankings of different indices used to uncover this hierarchy.)

At the second level of importance in distinguishing between these three morphotypes is a set of four indices (all ranking equally top place in PC2 contributions), pertaining to cilia characterization. While the first two of these indices characterize cilia count, i.e., the density of cilia per Anthrobot (measured by “cilia points”), and the density of cilia per unit area of Anthrobot (measured by “cilia points/area”); the remaining two indices characterize the pattern in which these cilia are distributed: how tightly grouped the cilia are (measured by “polarity,”), and the number of “free‐floating” ciliary patches that are not within a group (measured by “noise points”). These four indices together describe type 2 bots as being significantly more ciliated than type 1 and type 3 bots, and type 3 bots as having a significantly more polarized cilia distribution pattern (with the least amount of extra‐cluster noise) in comparison to type 1 and type 2 bots (Figure 3D).

The third most important (scoring a second level rank in both PC1 and PC2) characteristic in distinguishing between the different morphotypes is a function of both the size/shape of the Anthrobot and the localization pattern of its cilia: the homogeneity of cilia distribution on the surface of the Anthrobot (measured by “cilia distribution homogeneity”). This local index is related to, but not directly anti‐correlated with, the polarity index, because while cilia distribution homogeneity characterizes local neighborhood patterns, polarity (along with its supporting index “noise points”) characterizes the global (entire Anthrobot‐level) cilia distribution. (See Experimental Section for more information). In this way, we obtain both a local and a global view of the cilia distribution patterns at once and identify type 1 bots as both globally and locally homogeneous, type 2 bots as globally homogeneous but locally heterogeneous, and type 3 bots as both globally and locally heterogeneous with high degree of global polarization.

In summary, our morphological characterization pipeline suggest that Anthrobots self‐organize into 3 major morphotypes (Figure 3E) and this relationship can be represented by a developmental decision tree shown on Figure 3F wherein the first “decision point” determines the Anthrobot size and shape. Accordingly, bots that are small and regularly shaped (morphotype 1) form one branch, whereas bots that are larger and more irregularly shaped (morphotypes 2 and 3) form the alternating branch. On this alternating branch a second decision point forms further downstream and determines Anthrobot cilia pattern. Anthrobots with a non‐polarized cilia pattern form one branch (morphotype 2), and Anthrobots with a polarized cilia pattern form the other (morphotype 3).

Finally, one characteristic that does not seem to be changing in any significant way between these three morphotypes is the ratio between the longest and shortest distance within a spheroid (measured by “aspect”). Although the 3 morphotypes differ significantly in terms of the volumetric regularities (measured by the shape smoothness index) as explained above, their aspect ratios are statistically very similar.

### 2.4 Distinct Movement Types and Morphotypes are Highly Correlated

Having observed the emergence of several discrete types of movement (Figure 2) and morphology (Figure 3), we next decided to investigate whether there is a mapping between Anthrobots’ different movement types and morphotypes. To do this, we incorporated an additional level of movement‐type information into the PCA analysis used for identifying the morphotypes as introduced in Figure 3. During the initial sample collection process for this analysis, we had been able to definitively distinguish between non‐motile Anthrobots (non‐movers) and motile Anthrobots (movers) as described in Figure 1C. To further represent the movement types observed within the mover population, we randomly sampled from the set of motile subjects, targeting 30 Anthrobots that translocated (i.e.*, displacing movers)* to assign a movement type. Selected displacing movers were randomly collected from the two most orthogonal movement types, circulars and linear, in approximately equal proportions.

**Figure 4:**
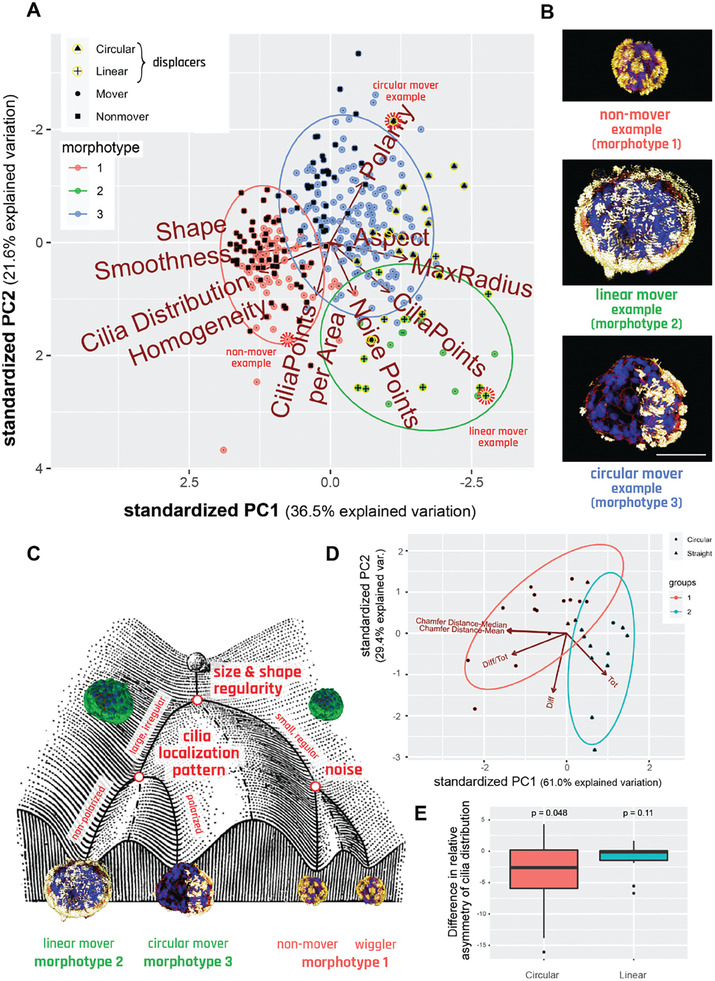
Distinct movement types and morphotypes are highly correlated. A) PCA of 350 bots forming 3 morphotypical clusters, showing that there is significant overlap between these clusters and the separately marked non‐movers, linear and circulars. Red dashed circles mark specific examples featured in panel B, selected from the cluster edges for distinct representation. B) Sample morphotype examples from each cluster, corresponding to Cluster 1, 2 and 3 and Nonmover, Linear, and Circular, respectively chosen for their ability to best represent the morphotype versus movement type mapping. Scalebar 50 uM and applies to all three bots. C) Waddington landscape illustrating the logic of determination of bot behavior and their relation to morphotypical indices with end behavioral products, as well as the potential states possible at each level of bifurcation of the bots’ development. (Waddington Landscape image modified from J. Ferrell, 2012.) D) PCA and unsupervised clustering showing the polarization among linear and circular bots in respect to bilateral symmetry metrics. E) Difference in asymmetry of cilia distribution between the movement axis and its 90‐degree offset axis. Here, n = 15 and 13 for Circulars and Linear respectively, with p = 0.0482 and 0.1116 done by one‐sample t‐test for each with alternative hypothesis.

Next, we identified these non‐mover and displacing mover Anthrobots within the PCA cloud presented in Figure 3C and assigned them this additional layer of information, i.e., movement type (**Figure** **4**A) without changing anything else in our sample pool or analysis workflow. In result, 62% of non‐movers were identified within morphotype 1 cluster (with the remaining 38% falling into the morphotype 2 cluster). A 100% of displacing bots were identified in morphotype clusters 2 and 3, with ≈85% of linear bots being in cluster 2, and 88% of circular bots being in cluster 3. We have further computed the statistical significance of these overlaps (using the Fisher test, see Materials and Methods) and conclude that the non‐movers, linear, and circulars significantly correspond with the morphotypes 1, 2 and 3, respectively.

The fact that none of the displacing bots were identified within the morphotype 1 cluster suggests that the movers identified within this cluster (i.e., those morphotype cluster 1 data points which are not labeled as “non‐movers”) are statistically likely to be *non‐displacing movers*, displaying a stationary wiggling motion. Accordingly, we conclude that morphotype 1 bots are likely to assume either non‐mover behavior or wiggler behavior. This may be attributable to their spherical shape with homogeneously distributed cilia where the propulsion forces generated by the ciliary motion are more prone to canceling each other out due to the radially symmetric spherical shape, resulting in little or no movement. Accordingly, inherent noise in the system (such as small imbalances in the cilia distribution on the spheroid surface or how the bot happened to be oriented in the plate) may be sufficient to have these bots generate small amounts of movement, causing them to wiggle, but not enough movement to become a displacing bot.

As a result of these analyses, we conclude that there is a statistically significant relationship between the Anthrobots’ developmental morphology and their behavior, and we show visual examples of this relationship with categorical examples on Figure 4B. We further represent this relationship by a decision tree in the form of a Waddington Landscape – a formalism often used to characterize cell‐ and body‐level properties by mapping out the sequential logic of decision‐points in transcriptional space or morphospace.^[^ ^24^ ^]^ Figure 4C shows the Waddington Landscape for the Anthrobot. The single cell at the top of the diagram represents the single cell that will develop into the multicellular Anthrobot. During this process of self‐construction, the Anthrobot moves through the developmental landscape, negotiating certain points of morphological possibility to reach its final architecture. We conclude that the unique and spontaneous 3D multicellular morphogenesis of adult airway cells into Anthrobots is consistent; the final form of the Anthrobot displays a degree of variability and exhibits discrete characters with easily recognizable primary features that also map on to phenotypic behavior.

### 2.5 Anthrobots Show Bilateral Asymmetry Along Movement Axis

The above metrics all focused on the global structure of the bot. Next, we studied the local characteristics that connect the movement of bots to their morphology, by looking for a difference in bilateral symmetry, or lack thereof, between the two major types of displacing bots (linear and circulars) through symmetricity measurements across plane coincident with their direction of movement. One hypothesis is that Anthrobots have bilateral symmetry that underlies their ability to move in straight lines (as observed in many existing species^[^ ^25^ ^]^ and even synthetic forms^[^ ^26^ ^]^); this hypothesis predicts that Anthrobots with circular motion should have more asymmetry across their movement axis compared to other planes. This hypothesis was tested by running a PCA and unsupervised clustering algorithm on a point cloud quantifying Anthrobot cilia distribution patterns through four major bilateral symmetry‐related measurements: total cilia points on a given bot (measured by “tot”), difference in number of cilia points between the two hemispheres (halves of the bot that are separated by the movement axis) of a given bot (measured by “diff”), this difference normalized by total cilia points (measured by “difftot”), and finally the bilateral symmetry index along the movement axis (measured by “Chamfer distance,” see methods for more details). Results of this analysis (Figure 4D) yielded two major clusters that, in a statistically significant manner, each correspond to one of the two major types of displacing bots (linear and circulars). The group consisting predominantly of linear bots scored significantly higher on the bilateral symmetry measurement (via Chamfer Distance axes, which inversely correlate with bilateral symmetry measurement). This result provides preliminary evidence in support of the hypothesis that Anthrobots with linear movement trajectories may have higher degrees of bilateral symmetry.

In order to further test this hypothesis, while also controlling for the globally homogeneous cilia distribution in linear bots posing a potential confounding factor for bilateral symmetry measurements, we compared the bilateral symmetricity of linear and circular bots against arbitrary axes other than the axis of movement. The initial hypothesis that linear Anthrobots may have higher degrees of bilateral symmetry compared to circular bots automatically suggests that for linear bots, we would expect there to be no other axis than the axis of movement along which the bilateral symmetry is higher; and for circular bots, we would expect there to be other axes than the axis of movement in respect to which the bilateral symmetry is higher. We tested this postulation by measuring linear and circular Anthrobots bilateral symmetry indices separately along each bot's axis of movement versus its farthest rotated (i.e., 90‐degree rotated) counterpart (as the control axis). As a result (Figure 4E), we indeed observed that while for the linear bots there exists no other axis in respect to which the bilateral symmetry is higher than that of the axis of movement, for the circular bots, there exists other axes than the axis of movement in respect to which the degree of bilateral symmetry is higher (p = 0.048). (See Figure S8, Supporting Information for comparison with other control axes that have rotational angle smaller than the farthest possible 90‐degrees.) Taken together, these findings support our hypothesis that Anthrobots with distinct movement types have distinct local bilateral symmetry profiles, with linear bots showing higher bilateral symmetry. This suggests that these synthetic forms recapitulate a fundamental morphological property observed in many wild‐type species.

### 2.6 Anthrobots Can Move Across Scratches on Live Monolayers In Vitro

One possible use of these living biobots is to manipulate other tissues, in vitro or in vivo, in future biomedical or bioengineering applications. How will biobots react to environments different from those that their component cells face in their native configuration in vivo? Thus, Anthrobot behaviors need to be characterized outside of a bare culture dish context, and especially in environments that airway epithelia do not normally encounter. Having characterized their baseline movement and morphology, we wanted to assay this novel motile form for potentially useful behaviors and ways in which it may interact with other somatic tissues, especially sites of damage. Because we are interested in surprising examples of behaviors in such novel constructs, we sought to confront them with a scenario which would not be natural for these airway cells, either in vivo or in their evolutionary history. We decided to study the ability of Anthrobots to move across live tissues that have been damaged, taking advantage of a common model system: the monolayer scratch assay in vitro.^[^ ^27^ ^]^ We produced 2D confluent layers of human neurons derived from human induced neural stem cells (hiNSCs) based on a previously established method,^[^ ^28^ ^]^ and introduced a scratch of 400–1000 microns by mechanically scratching away the neuron layer in a long swath. We chose hiNSC‐derived scratches, instead of (for example) smooth and regular polydimethylsiloxane (PDMS) channels, because complex borders of such in vitro live tissue scratches featuring live cells constitute more biologically realistic in vitro proxies. We are interested in developing aspirational models for more complex scratched multi‐layered live tissues which are more prone to reveal novel and interesting interactions compared to gels or other artificially‐smooth surfaces.

**Figure 5:**
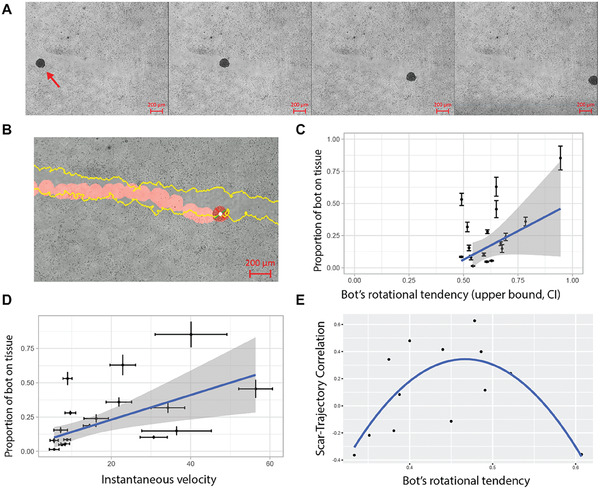
Anthrobots can move across living monolayers in vitro. A) A representative timelapse video of an Anthrobot as it moves along a neural scratch in vitro. B) Sample tracking video output with scratch edge highlighted in yellow and bot path in red. The rotation of the bot is measured through the change in the orientation of the green and red bars attached to the center of the bot in white. C) The significant (p = 0.017, slope 1.15, n = 17, t‐test for slope) positive relationship between bot gyration index and proportion of bot's body in contact with scratch suggest that circular bots cover the edges of the scratch more as they move along the scratch. D) The significant (p = 0.031, slope 0.0082, n = 17, t‐test for slope) positive relationship between bot speed and proportion of bot's body in contact with scratch further suggest that faster bots also cover the edges of the scratch more as they move along the scratch. E) For a subset of bots (dataset constrained to non‐stalling bots with rotational tendencies between 0.33 and 0.7 and viable tracking videos), the quadratic (alternative curves were insignificant) relationship (p = 0.006, n = 13, t‐test) between bot gyration index and scratch‐trajectory similarity metric suggests that there is a goldilocks zone for the bot rotational tendency for maximum scratch area exploration. This quadratic relationship was revealed when we initially tested for a linear relationship between these two metrics by plotting the residuals against the fitted values for the model, and observed a clear quadratic trend among the residuals (see Figure S9, Supporting Information), which strongly suggested the fitting of a quadratic model instead, which is shown here. Consistent with these statistical analyses, in the experimental space we observed that bots with very low rotational tendencies interacted minimally with the scar edges while bots with very high rotational tendencies skidded in place or was prone to veering off the scratch edge. There appears to be an optimal amount of rotation for a bot to move across the scratch while faithfully following the scratch edge.

Anthrobots were placed within these neuronal scratch environments in order to characterize their dynamics in this novel biological environment. Bots were allowed to freely move on their own and timelapse videos were recorded (**Figure** **5**A, and Videos S6 and S7, Supporting Information). These videos were then tracked, and specific indices were calculated from the tracked files (Figure 5B). Among these indices, we characterized the degree to which bots interact with the native tissue surrounding the scratch (measured by “proportion of bot on tissue”), bots’ tendency to assume a circular motility profile (measured by “bot's rotational tendency”), and bots’ displacement speed in traversing the scratch (measured by “instantaneous velocity”). More specifically, we investigated the relationship between bots’ efficiency in traversing the scratch as a function of the circularity of their movement pattern (Figure 5C). We observed a significant positive relationship (slope = 1.5, p = 0.01), confirming our baseline assumption that although circling bots are less efficient in forward motion, they are better at covering the most unique coordinates in the scratch. We have further observed that the instantaneous velocity also had a significant positive relationship with bots’ efficiency in moving across the scratch (slope = 0.0082, p = 0.031 (Figure 5D), presumably due to increased collisions with the tissue. Taken together, these data reveal that Anthrobots are capable of efficiently moving across damaged tissues, and that bots that have a higher rotational tendency and or higher speed have higher degrees of unique coordinate coverage by moving across a higher percentage of the scratch interface.

Having observed the behavior of these bots in scratches, we focused on the interactions between various scratch edge patterns and bots’ ability to track along them. In order to do so, we first constrained the dataset to trajectories that could be used to further understand this relationship. We specifically focused on bots that were not extreme in their rotational tendencies, had ample contact with the scratch and had viable tracking videos (see Experimental Section). This enabled us to isolate the tendency of the bot to turn consistently in the same direction (measured again by “bot's rotational tendency”) and the correlation between the trajectory and scratch edge (measured by “scratch‐trajectory correlation”). With our constrained dataset, we saw that gyration had a quadratic effect on scratch‐trajectory similarity (Figure 5E) with p = 0.006, suggesting that there is a specific range for gyration where the scratch‐trajectory correlation can be maximized: Anthrobots can be chosen to specifically maximize coverage efficiency based on their rotational tendency.

### 2.7 Anthrobots Can Promote Gap Closures on Scratched Live Neuronal Monolayer Tissues

One of the most important aspects of exploring synthetic morphogenesis is the opportunity to observe novel behaviors that are obscured by standard, default phenotypes. Having seen that these airway cell‐derived constructs can move along and settle in neural scratches, we decided to check for the effects of their presence on the surrounding cells. A characterization of their wild‐type capabilities is important not only for understanding biological plasticity but also for establishing a baseline for future efforts in which biobots are augmented with additional synthetic circuits for pro‐regenerative applications.

**Figure 6:**
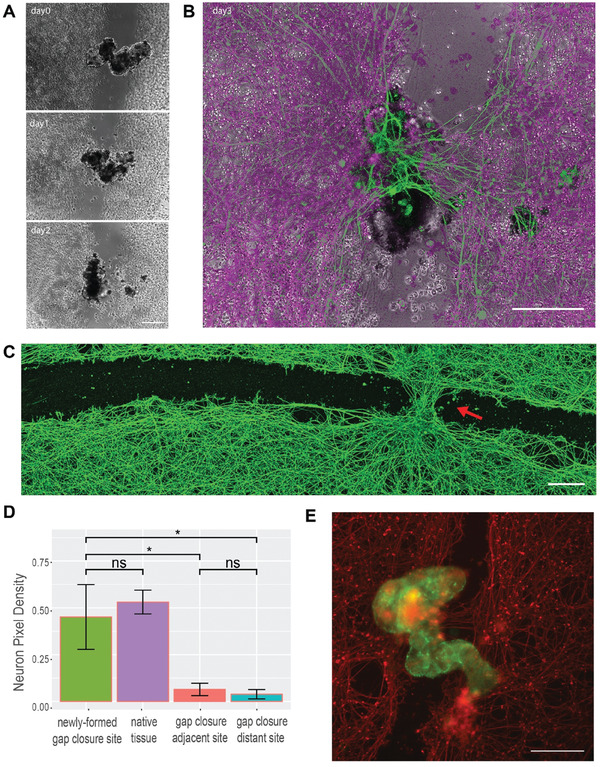
Anthrobots can promote gap closures on scratched live neuronal monolayers. A) sample micrograph of a bridge across a neural scratch over time from bridge inoculation day (day0) to days 1 and 2. B) An overlay of a bridge bot and the induced “stitch” (i.e., gap closure site) at the end of the observation on day 3. C) Immunological staining of neurons with Beta III Tubulin (Tuj1) upon fixation on day 3 after the bots were introduced to the system, showing an induced neural gap closure at the site of bot settlement. D) Among N = 10 experimental replicates, 50% of the Anthrobot bridges have maintained connectivity to both sides of the scratch area across all three days of the experiment (i.e., fully connected bridges), and among these bridges, 100% has yielded gap closure underneath at the neuronal scratch site. Shown here is a quantification of the resulting tissue on day 3 of all fully connected bridges measured by average proportion of neuronal coverage by pixel counts for each positional category: gap closure site, unscratched native tissue (calculated by the average of the two neuron‐heavy area pixel coverage), adjacent and distant sites to the gap closure. Difference between gap closure site and native tissue is insignificant (p = 0.37), while the difference between the gap closure site and both adjacent and distal scratch sites are significant (w/ p = 0.006 and p = 0.005, respectively); that suggests the tissue at the gap closure site is as dense as the native tissue, and the gap closure effect follows a crisp profile as opposed to a gradient profile. P‐value range of 0 to 0.0001 corresponded to ****, 0.0001 to 0.001 corresponded to ***, 0.001 to 0.01 corresponded to **, 0.01 to 0.05 corresponded to * and 0.05 to 1 corresponded to ns. See methods section for example frame of a sampling region. Scratch lengths varied between 150–500 um E) Immunological staining of another sample bridge superbot (green) and the neuronal tissue (red). All scalebars on this figure feature 200 microns.

Inspired by collective behavior and swarm intelligence, and more generally, how in nature collectives can accomplish tasks that individuals cannot, we decided to create “superbot” assemblies by facilitating random self‐aggregation of distinct Anthrobots that fuse to form larger structures. We accomplished this without using molds or any other external shape‐giving equipment, but by simply constraining multiple Anthrobots in a relatively smaller dish, while keeping everything else constant. Akin to how ants cross openings that are too wide for a single ant to cross by forming a bridge through aggregation of their bodies,^[^ ^29^ ^]^ we placed these superbots into arbitrary sites along the tissue scratch such that they span the entire width of the scratch, enabling them to “bridge” two sides of the damaged tissue in order to see if we can induce any kind of repair of the scratched monolayer by bridging the two sides, akin to a mechanical stitch. **Figure** **6**A shows a superbot on a scratch upon its placement on day 0, as well as the resulting bridge configuration on subsequent days of day 1 and day 2.

Strikingly, within the next 72 h upon inoculation of the superbot into the tissue scratch on day 0, we observed a substantial regrowth of the native tissue taking place (i.e., gap closure), resulting in the formation of a stitch right underneath the “superbot bridge,” connecting the two sides of the scratch (Figure 6B). This gap closure was observed solely at the site of superbot inoculation, and at no other place along the long scratch (Figure 6C). A quantitative analysis (Figure 6D) of these gap closure sites shows that while the neuron pixel coverage density of the gap closure site is as high as the native tissue outside the scratch (see Figure S10, Supporting Information), the rest of the scratch space, whether adjacent or far, had significantly less density of coverage. Thus, the density of the induced gap closure area that formed as a result of the presence of the superbot represented full (statistically indistinguishable from 100%) recovery of the original tissue and was uniquely different from the surrounding scratch area. Further quantification of superbot bridge‐assisted neuronal gap closure formation showed an average aspect ratio of 0.7 on average. Finally, in order to test whether simple (passive) mechanical contact was sufficient to induce the same effect, we incubated neuronal scratches for 4 days with a piece of agarose on top to provide mechanical loading; this induced no repair (see Figure S11, Supporting Information).

## 3 Discussion

Biorobotics and bioengineering have at least two main areas of impact. One is the production of useful living machines.^[^ ^2a–c^ ^]^ The other is the use of unconventional configurations for living materials at all scales, to probe the macro‐scale rules of self‐assembly of form and function.^[^ ^3^, ^30^ ^]^ Specifically, by confronting evolved systems with novel contexts, we can learn about the degree of plasticity that cells and control pathways can exhibit toward new anatomical and functional endpoints, as well as develop protocols to alter default outcomes. Here, we used human patient cells to begin the journey toward immunologically‐acceptable, active, living biomedical constructs, and to begin to probe the morphological and functional capabilities of mammalian, adult cells.

Self‐motile, fully‐organic biobots have been demonstrated with frog cells^[^ ^7^ ^]^; however, it was unknown whether the surprising properties of Xenobots depend strongly on their amphibian genome and evolutionary history, as well as their embryonic state. Specifically, the plasticity of amphibian tissues, and the propensity of embryonic cells to self‐assemble into structures were thought to be unique features that may not be available to engineers working with adult patient‐derived cells. We show that despite spending their entire life in a flat, tracheal architecture (a cycle of over 4–8 decades for our donors), these human cells, with a wild‐type genome and no introduction of scaffolds or nanomaterials, are able to implement a novel set of morphogenetic classes and motile behaviors. Another surprising finding, given the usually tight mapping between genomes and species‐specific form and function, is that the Anthrobots adopt some of the morphological and functional properties similar to Xenobots despite their highly divergent genomes.

Anthrobots’ overall shape and behavior are similar to that of Xenobots, but not identical. Anthrobots are ≈20 to 300+ micrometers in size, whereas Xenobots range from an average of 487±39 µm for the smallest cut explants to 602±30 µm for the largest^30^. Xenobots likewise offer discrete motion types, and their behavior transition profile is similar. The interconversion between linear and circular is very small for Xenobots (0.5% and 1.6%) and Anthrobots (0.2% and 0.3%), while their consistency of Circular behavior is extremely high for Xenobots (95%) just like for Anthrobots (92%). However, Xenobots’ linear behavior consistency was not as high (67%) when compared to Anthrobots (80%). Despite their highly divergent genome, age, and tissue origin, the two platforms assemble into very similar types of creatures, illustrating the importance of generic laws of morphogenesis^[^ ^31^ ^]^ in addition to species‐specific genomic information.

Another difference from existing biorobotics is that the Xenobots’ construction depended on a rate‐limiting process of extracting source cells from frog embryos. Here we show a protocol for enabling the self‐construction of Anthrobots: living structures made from epithelial cells that traverse aqueous environments. The process is highly scalable, and produces Anthrobots in the course of 3 weeks, with minimal manual input beyond weekly media changes. At the end of their 4–6 weeks life span, they safely degrade by becoming unviable debris.

Anthrobots exhibit several distinct movement and morphological classes, which are significantly correlated. This is especially important because the structure and function of this novel construct is not that of a familiar organism (despite a wild‐type genome), and it was not yet known whether its morphospace possessed specific attractors, how reliable the cells’ navigation of that morphospace was, or how the movement patterns would relate to its specific morphology. Anthrobots showed clear and consistent active movement types, quantified over 30 second periods: circulars, linear, curvilinear, and eclectics, with the last category including the non‐displacing bots, i.e., wigglers, as well as distinct morphotypes that are best distinguished by Anthrobot size, shape, and cilia localization patterns. While more work needs to be done to establish a causal relationship between these morphotypes and the movement types, our analyses showed significant correlation between the non‐displacing (wiggler or non‐mover) movement type and morphotype 1, linear movement type and morphotype 2, and finally circular movement type and morphotype 3. Such correlation has implications for future control of higher‐order behaviors (such as movement types) by way of controlling Anthrobot morphology through synthetic morphogenesis, as well as real‐time physiological signaling. In the future, machine learning classifiers may help predictively identify different movement types from phase contrast images of live bots, without needing to perform immunostaining on them. Furthermore, such classifiers will use artificial intelligence tools to correlate initial physiological parameters with final outcomes, as part of the effort for using Anthrobots as a platform for cracking the morphogenetic code.

Analysis of movement and morphology has further revealed the ability of the Anthrobots to establish bilateral symmetry, which is an interesting aspect of self‐assembly in a symmetrical environment and will enable future studies of the still poorly‐understood question of how multicellular amniote embryos bisect themselves to establish a single midplane for their bodyplan.

We found that Anthrobots can traverse neural tissues and defects therein. This popular but highly simplified injury model^[^ ^27a^ ^]^ is just the beginning for understanding how Anthrobots will deal with traversing complex multifaceted 3D tissues. Most remarkably, we found that Anthrobots induce efficient healing of defects in live human neural monolayers in vitro, causing neurites to grow into the gap and join the opposite sides of the injury. Passive materials did not recapitulate this effect, but it is not yet known which of the many possible biochemical and biophysical aspects of Anthrobot presence are required for this. Although the complex in‐vivo dynamics (e.g., immune components, migratory cells, inflammatory signaling and so on) that may otherwise be observed in actual wounded tissues are not present in this simplified in‐vitro neuronal injury model, so are the endogenous repair cues (e.g., chemical gradients that normally guide such repair processes), yet the Anthrobots were still able to facilitate the repair of a scratched neuronal monolayer. Future work will examine the functionality of Anthrobots in complex injury sites in vivo and identify which of their properties and active processes are mediating the effects.

Furthermore, the size of the Anthrobot to facilitate this repair can be adjusted. The tendency of the bots to fuse together and thus form different sized collectives (i.e., superbots) for different scratches can be controlled by modulating the number of Anthrobots cultured together in the same well for fusion, which happens only on days immediately following dissolution while the Anthrobot basal layers are still exposed and thus can mediate the bot‐to‐bot fusion. The finding is unexpected given these tissues’ normal roles in the human body – the fact that wild‐type cells from trachea will move over and heal neural tissues could not be predicted from any current molecular or tissue‐level models. Thus, it is likely that screens for engineered interactions between body tissues in the context of motile bio‐robotics and other preparations should be performed to uncover novel capabilities of cells and multicellular constructs. Likewise, future molecular biophysics and machine learning efforts could identify the specific signaling modality that is used by Anthrobots to induce neural repair in their vicinity, and thus harness this effect for therapeutic purposes.

Anthrobots are derived from adult human tissue, and in the future could be personalized for each patient, enabling safe in‐vivo deployment of these robots in the human body without triggering an immune response. Once inoculated in the body via minimally invasive methods such as injection, various applications can be imagined, including but not limited to clearing plaque buildup in the arteries of atherosclerosis patients, bulldozing the excess mucus from the airways of cystic fibrosis patients, and locally delivering drugs of interest in target tissues. The possible applications will represent those arising from exploiting surprising novel behaviors of cells and engineering new ones via future synthetic biology payloads, such as novel enzymes, antibodies, and other ways to manipulate the cells they traverse and interact with. They could also be used as avatars for personalized drug screening,^[^ ^32^ ^]^ having the advantage of behavior over simple organoids, which could be used to screen for a wider range of active, dynamic phenotypes.

## 4 Conclusion

We quantified in detail the morphogenetic and behavioral capacities that self‐organized, clonally‐derived biobots can develop in culture from adult, genetically wild‐type, human cells. We found correlations between their specific form and several modes of autonomous (self‐driven) motile function, and characterized the space of discrete characters of form and function that are not currently inferable from the standard target morphology associated with the human genome. We also found a surprising non‐cell‐autonomous functionality of Anthrobots – a repair property that likewise could not have been guessed in advance from existing frameworks describing the uses of organoids and other bioengineered structures.

Anthrobots may be able to be generated from other ciliated cells in the human body (e.g., oviductal epithelia or brain ependymal cells), and other cell types can be used for bots when autonomous motility is not needed (they can still perform various functions such as the healing we report here, or sensing/reporting, etc.). It may also be possible to induce a ciliation program in other body cell types. These data establish a research program with many unanswered questions for subsequent work. What other cells can Anthrobots be made of? What other behaviors might they exhibit, and in what environments? What other tissue types can they repair or affect in other ways? Can transcriptional or physiological signatures be read out in living bots, that reflect their past and immediate interactions with surrounding cellular or molecular landscapes? Do they have preferences or primitive learning capacities,^[^ ^33^ ^]^ with respect to their traversal of richer environments? More fundamentally, these data reveal additional morphogenetic competencies of cells which could have implications for evolutionary developmental biology, as evolution of anatomical and functional features could be affected by the ability of the same genome to produce very diverse forms in different environments. Finally, this kind of new model system is a contribution to two key future efforts. The study of synthetic biological systems^[^ ^3^, ^34^ ^]^ is an essential complement to the standard set of phenotypic defaults available in the natural phylogenetic tree of Earth, revealing the adjacent possible in morphological and behavioral spaces.^[^ ^35^ ^]^ Moreover, these systems offer a safe, highly tractable sandbox in which to learn to predict and control the surprising and multi‐faceted system‐level properties of multiscale complex systems.

## 5 Experimental Section

### Production of Anthrobots via NHBE Culture

NHBEs were sourced from Lonza Walkersville, MD (CC2540S). The cells were first thawed and seeded on a T150 flask containing bronchial epithelial growth medium (BEGM, Lonza CC‐3170) for 2D cell culture growth. Once the NHBEs were ≈80% confluent, they were passage into a 24‐well‐plate of Matrigel (Corning #354 230) beds for 3D cell culture. The NHBEs were not passed past the 3rd passage. Each Anthrobot bed contained 500 µL of 25% Matrigel, 0.1% 0.5 nM retinoic acid (Sigma–Aldrich R2625) and in bronchial epithelial differentiation medium (BEDM, which is 50% BEGM without T3 and 50% high glucose Dulbecco's Modified Eagle Medium (DMEM) without Sodium Pyruvate from Sigma #11‐965‐092) that was centrifuged for 5 s at 100 x g and prepared at least 4 h before passaging the cells. We usually made 6 beds, but this number can be adjusted at discretion. The cells were re‐suspended in 5% Matrigel, 95% BEDM and 0.1% 0.5 nM Retinoic Acid (RA) and seeded directly onto the Matrigel beds with 500 µL per well at a 30 000 cells mL^−1^ concentration. Once seeded, the NHBEs were centrifuged for 5 s at 50 x g. On days 2 and 8, the NHBEs received a top feed containing 750 µL 5% Matrigel, 95% BEDM, and 0.1% RA. On day 14, 500 µL of the wells’ contents was aspirated and 500 µL of dispase (#D469) at concentration 2 mg mL^−1^ was added to each well. A mini cell scraper was used to break up the Anthrobot clumps and then followed by a 0.05% Triton coated pipette tip to mix up the Anthrobot with the dispase. The dispase was then incubated at 37 °C for 1 h with the pipetting process repeated every 15 min. During incubation, Pluristrainer Mini's with a 40 µm pore size (Fisher Scientific #431 004 050) were placed in wells of a fresh 24‐well‐plate that contained 2.5 mL of 0.05% Triton. After the incubation period, 250 µL of 1% 5 mM EDTA in Dulbecco's Phosphate Buffered Saline (D‐PBS) was added into each well. The media in each well was then drawn up, using the Triton‐coated tip, and added to the Triton‐coated strainers. The NHBE spheroids in the strainer were rinsed with 1 mL of D‐PBS then expelled onto a low adhesive dish by inverting the strainer over the dish and expelling 5 times of 1 mL of BEDM through the bottom of the strainer. After all spheroids were in one dish, they were divided evenly among multiple 60 mm dishes by using a Triton‐coated pipette tip and a microscope to manually draw up and divide them. 0.5 µL of 0.5 nM retinoic acid was added into each dish once divided. For the next 14 days as the spheroids started moving, they required 0.5 µL of 0.5 nM RA every other day and a media change every 4 days. The media change was performed by swirling the Anthrobots to the center of the dish then collecting 2 mL of old media and adding 3 mL of fresh BEDM.This was done under a microscope to ensure no Anthrobots got aspirated. Finally, it was created “superbot” assemblies by facilitating random self‐aggregation of distinct Anthrobots that fuse to form larger structures. It was accomplished this by transferring one well's equivalent of Anthrobots into a 60 mm dish, while keeping everything else constant.

### Tracking Timelapse Videos

Timelapse videos of the Anthrobots were collected at 2.5 s intervals for a duration of 5 h. The videos were contrast‐enhanced using the video editing software ImageJ for optimal tracking and data that are within one bot length (≈100 um) from the edge of the vessel were omitted to prevent edge effect as a confounding factor. They were then processed to extract the trajectories of the Anthrobots utilizing the trackR function in the trackR package (version 0.5.1) for R developed by the Swarm Lab of New Jersey Institute of Technology (NJIT). The software parameters were chosen manually in order to increase the accuracy of the tracking, following the instructions in the trackR package's help. Tracking errors such as the swapping, deletion or insertion of tracks were subsequently manually corrected using the trackFixer function from the same package.

### Movement Type Analysis

From the extracted trajectories, the following metrics were computed: i) the linear distance between the current position and the immediately preceding one; ii) the linear speed at each position, approximated as the distance moved between the current position and the immediately preceding one during the time interval between these two positions; iii) the heading of the bot at each position, approximated as the angle between the vector formed by the current position and the immediately preceding one and that formed by the Anthrobot position and the immediately following one; iv) the angular speed of the bot at each position, approximated as the difference between the heading at the immediately preceding position and that at the current one during the time interval between the corresponding three positions required to calculate these two headings; v) the time difference between each position.

Behavioral classification was then performed on non‐overlapping 30 s blocks of trajectory. To determine how predictable a position change was, the linear speed, heading, and angular speed were estimated at each position to predict the coordinates of the following position. The error (Euclidean distance) between the predicted coordinates and the actual coordinates was then computed. For each complete 30 s block of trajectory (i.e., a block with no missing timestamp), total error over the entire block was calculated and normalized by the total distance traveled during that block to account for the artificial error amplification caused by predicting over longer distances. To separate active from inactive blocks, an automated classification method was used on the distribution of total normalized errors. A gamma mixture model with two components was fit to the data using the expectation maximization algorithm in the REBMIX function from the rebmix package (version 2.12.0) for R.^[^ ^36^ ^]^ The 30 s periods in the resulting cluster with the highest total normalized error were considered as inactive and excluded from further classification.

We then derived two metrics to describe each trajectory: i) a “straightness” index computed as 1 minus the circular variance of the headings during the block (a value of 1 indicates a perfectly straight line) and ii) a “gyration” index computed as 1 minus the circular variance of the angular speed during the block divided by the circular variance of the same angular speeds and their additive inverse, which helps in taking into account the magnitude of the angular speeds themselves (a value of 1 indicates a trajectory following a perfect circle).

The need for two indices arises from the fact that a straightness index alone cannot fully tease apart all different movement types due to its aggregate view of a trajectory. In other words, a low straightness index does not automatically translate into a perfectly circular bot, as we can see in Figure S6A.1,A.2 (Supporting Information) with the arc trajectory. This is due to the fact that the straightness index does not account for the time‐dependent dynamics and thus ignores individual variations across frames. This is where the second movement metric, the gyration index, comes into play. To account for temporal information, we calculate the angular speed, which is the difference between successive headings divided by time between frames and thus has units of radians/ second. Figure S6B (Supporting Information) shows a bent trajectory and Figure S6C (Supporting Information) shows an arc trajectory, both of which have similar straightness indices. However, when we start looking at their temporal relationships using angular speeds, the behavior is entirely different. For the arc (Figure S6C, Supporting Information), the variance of the angular speed is very small since the change in heading of the trajectory each time is relatively consistent (the distribution shown in the histograms).

For the bent trajectory (Figure S6B, Supporting Information), the variance of angular speed is much larger than the arc since for most of the trajectory the angular speed is close to 0 (it goes straight), but the bent portion has a very high angular speed, i.e., the angle changes very quickly. In general, the greater the absolute value of the angular speed the sharper the turn in the trajectory (zero is straight) and the greater the variance of the angular speeds, the less the consistency of the turns in the trajectory. A circle or arc usually has absolute values of the angular speed much greater than zero and low variation of angular speed. However, the gyration index alone cannot differentiate between all behavior either. Let's look at a circular trajectory (Figure S6D, Supporting Information). Even though the absolute values of the angular speeds between arcs and circles are different, the circle also ends up having a gyration index close to 1 since all the turns in a circle are highly consistent like in an arc and thus the variance of the angular speed for both is very small. This fact means the gyration index cannot segregate between arcs and circles, among other things, by itself. Interestingly, the straightness index is exceptional at separating arcs and circles. This shows that though either index alone cannot distinguish all movement types well, together they can accomplish much more.

To separate the trajectory blocks into categories of similar behavior after calculating the movement metrics, a cross‐entropy clustering algorithm was used,^[^ ^37^ ^]^ and implemented in the cec function of the CEC package (version 0.10.2) for R.^[^ ^38^ ^]^ This yielded us six categories, of which trajectories from categories numbered 3 and 4 were merged into categories numbered 1 and 2 respectively due to the difference being phenotypically minimal. In the “behavioral space” as defined by the straightness and gyration indices, cluster 3 had the same straightness index range as cluster 1 and a lower gyration range between roughly 0.65 and 0.95, which represented trajectories that were highly circular but fell short of cluster 1 which represented “prototypical circulars”. Similarly, Cluster 4 had a slightly smaller straightness index range than cluster 2 (0.7 to 1 instead of 0.6 to 1) and higher gyration range between 0.1 and 0.55 which represented trajectories that were mostly linear but did not have a high enough gyration to be curvilinear or low enough gyration to be Cluster 2, a “prototypical linear”. The merge of the two clusters increased the average dissimilarity of the cluster, but it is a testament to how similar clusters 1 and 3, and 2 and 4 were already that their dissimilarity still remains very low at ≈0.09 and ≈0.14 respectively. Last, to understand how the bots’ behaviors are distributed relative to each other, transition probabilities between each behavioral category were estimated by calculating the proportion of times a block of a given category is followed by a block of the same or another category. This was then presented in the form of a Markov Chain.

### Immunocytochemistry/Immunofluorescence

Anthrobots were collected in Pluristrainer Mini's with a 40‐micron pore size (Fisher Scientific #431 004 050) and fixed with 4% paraformaldehyde at room temperature for 30 min. Following phosphate buffered saline (PBS) washes, blocking and permeabilization were performed for 1 h at room temperature on a rocker in a blocking buffer consisting of phosphate‐buffered saline with 10% normal goat serum, 1% bovine serum albumin (BSA), and .15% triton x‐100. Anthrobots were then incubated with mouse anti‐acetylated tubulin (Sigma‐Aldrich #T7451) primary antibodies at 1:250 dilution factor in blocking buffer for 24 h at 4 °C on a rocker. The primary antibodies were labeled with Alexa Fluor 647 donkey anti‐mouse (Thermo Fisher Scientific #A31571) secondary antibodies, at 1:500 dilutions in blocking buffer, for 1 h at room temperature on a rocker. Lastly, Anthrobots were incubated with Alexa Fluor 594‐conjugated mouse anti‐ZO‐1 (Thermo Fisher Scientific #339 194) at a 1:100 dilution in blocking buffer for 24 h at 4 °C on a rocker. Anthrobots were mounted on glass‐bottom 96‐well plates in ProLong Glass Antifade Mountant with NucBlue (Thermo Fisher Scientific #P36981). Neuronal tissues were fixed, blocked and stained using the same protocol, except by using Beta III Tubulin (Tuj1) (Abcam #ab18207) as the primary antibody for staining the hiNSCs. Images were collected using a Leica SP8 Fluorescence Lifetime Imaging Microscopy (FLIM) with a 25x water immersion objective. Z‐stack step size = 3 micron unless otherwise specified.

### Morphotype Analysis

To find whether there were any unique morphological types like was the case with movement types, each spheroid was processed through a custom‐made analysis pipeline (code attached). First, a 3D model of the bot was created in R where the points corresponding to the body and the cilia were clearly indicated. For this, first the cilia channel was isolated from the Laser Induced Fluorescence (LIF) images of the bots; these cilia‐only images were then run through CiliaQ^[^ ^39^ ^]^ using the RenyiEntropy algorithm for detection. These binarized cilia were then imported into the code, along with the points that comprised the body. These “body points” were extracted using the “body channel” of the LIF images by first running the pixels through a logistic transform then thresholding the pixels based on the signal to noise ratio, calculated by using a median filter and comparing the points before (“signal + noise”) to after (“only signal”).

Due to the large volume of body points, to reduce the points to a manageable amount, we first found the outlines of each slice of the spheroid by using a concave hull using the Concaveman package (version 1.1.0).^[^ ^40^ ^]^ The cilia points were then projected on the nearest body points by simply choosing the nearest one by distance to get the shadow of the cilia on the body.

The structural index variable Cilia Points was calculated by counting the number of unique projected points on the body. Then, the dbscan package (version 1.1‐10)^[^ ^41^ ^]^ function in R was used to find the clusters of cilia. The number of points that fell outside of clusters with this definition were defined as Noise Points.

Following this step, we computed the spanning ellipsoid of the body points by using the “ellipsoidhull” function from the cluster package (version 2.1.3).^[^ ^42^ ^]^ The Max Radius variable was calculated directly by the function, and Aspect was defined as the ratio of the largest radius to the shortest radius, all quantities computed by the function. Finally, we used the “ashape3D” function from the alphashape3d package (version 1.3.1)^[^ ^43^ ^]^ to generate a 3D alpha hull of the body points, and used the mesh to get the surface area of each spheroid.

Cilia Points/Area was defined as the Cilia Points variable divided by the calculated surface area. Similarly, the Shape Smoothness was defined as the ratio of the volume of the 3D alpha hull to the volume of the spanning ellipsoid. Finally, we found the center of the bot by finding the sum of the centroids of each triangle that makes up the alpha hull weighted by area of the triangle. Polarity was defined as the norm of the vectors from the center to each cilia point divided by the sum of the norm of each vector. The Cilia Distribution Homogeneity was defined as 1 – D statistic of the two sample Kolmogorov‐Smirnov test, where sample A is the 1st nearest neighbor (1NN) distances for the cilia, and sample B the 1NN distances if the same number of cilia points were distributed close to uniformly but randomly across the surface of the bot. After these analyses were carried out, we ran the dataset through a Principal Components Analysis with centering and scaling. Afterwards, a hierarchical clustering was carried out on the resultant dataset with the Ward.D2 method and the resulting classification was plotted as above. In total, 350 bots were put through the pipeline and into the following PCA and included Movers, Nonmovers, Linear and Circulars. Further details could be seen in the code.

To get a confidence interval for the absolute value of the loadings, we bootstrapped the loading value by sampling 250 bots from the 350 that we have, 10 times. We then took the loadings value for the 1st and the 2nd primary component for all 8 variables and calculated the mean and 95% Confidence Interval for the loadings for the PC in question. If there was overlap between the CI of the loadings, they were assigned the same rank, otherwise they were assigned different ranks. Ranks were relative to the “highest” contributor of the rank; i.e, for PC1, Shape Smoothness had overlap with Max Radius, but it also had overlap with Cilia Distribution Homogeneity. However, Cilia Distribution Homogeneity did not overlap with Max Radius. Max Radius had the highest upper limit of the CI among the 3 variables in question, and thus Shape Smoothness was co‐ranked #1 along with Max Diameter, but Cilia Distribution Homogeneity was not.

### Morphotype and Movement Type Overlap

After finding trends among behavioral and morphological data, we decided to see if there was any potential overlap between the two. To eke out any possible correlation, we first chose to use categories of spheroids behaviorally orthogonal to movers, the non‐movers. The goal was to observe whether there is any overlap between the morphology of movers and non‐movers. Similarly, we had four potential behavioral types that could overlap with our morphological clusters. Eclectics could not be included in the analysis since they are an aggregate of multiple inconsistent patterns and highly uncommitted to their behavior (Figure 2G), and thus cannot be used in a bot‐level analysis (instead of period). Circulars and Linears on the other hand were highly committed behavioral types that were orthogonal to each other (had little to no interconversion on the Markov plot) and prototypes of two extreme movement types with high variability between them. Consequently, the Curvilinear behavioral subtype was also not included since it lacked orthogonality with both Circulars and Linears due to shared traits between them. The morphological indices for each spheroid were calculated as outlined in the methods for the previous sections, and then clustered with Circulars, Linears, Nonmovers and Movers together. To measure the significance of the overlap, if any, between clusters, we decided to use a Fisher test to compute whether the proportion of a certain behavior per cluster type was different from the others. We ran the test twice, once to see if there were any significant differences in number of nonmovers per cluster, and once to compute the difference in the ratio between circulars and linear per cluster. It showed that the proportion of nonmovers in Cluster 1 versus Clusters 2 and 3 were significantly different with an average p = 2.6*10e‐6 and 3.5*10e‐8 respectively Cluster 2 and 3 also had a statistically significant difference in number of nonmovers (p = 0.01) which is understandable, since Cluster 2 had no non‐movers. For Circular/Straight, Cluster 2 versus 3 were significantly different with p = 0.00011, and Cluster 1 had no Circulars nor Straights.

### Motility Orientation Alignment and Movement Axis Analysis for Bilateral Symmetry

The generated tracks were analyzed alongside Z‐stacks of designated Anthrobot from a confocal microscope to see if their morphology was connected to their movement. ImageJ was used to compile the slices of the Anthrobot so that a 3D model could be generated and rotated to render a transformation that visually matches a random frame of the Anthrobot from the timelapse. This random selection could be done as the Anthrobot, despite moving around, did not tend to roll and therefore generally maintained the same orientation throughout a timelapse. Additionally, they often moved with a specific side that always faced forward that was designated as a heading. To see if biases in cilia patterns to one side or lack thereof on an Anthrobot affected its movement this heading would serve as the axis along which a plane of symmetry would be extended to bisect the bot. This plane would be defined by 3 points on the bot along this axis, one placed at the centermost point of the axis within the bot, one on the part of the bot that most visually served as the heading in the video and one on the opposite point of the bot from the heading.

### Bilateral Symmetry Along Movement and Other Axes

After realizing that polarity could play a key role in determining movement type, we decided to see whether the symmetry across the movement axis any trends had compared to the other axes. To calculate this, we followed the procedure used in both Figure 3 and Figure 4 to get representations of Cilia on the body of the bot, then project these representations onto the plane of symmetry defined by the points obtained in the Motility orientation alignment section. The side of the plane (movement axis) each cilia point belonged to was noted using the sign of the dot product of the normal of the plane and the vector to the cilia point. Finally, to better distinguish whether the cilia distribution played a role in movement type (linear vs. circular) we created the Bilateral Symmetry index. This index was modified from the Chamfer distance, and was calculated as the sum of the median/ mean of the distances between all points in set A and the closest point in set B and the median/ mean of the distances between all points in set B and the closest point in set A. To calculate the index, the cilia points were projected onto the plane defined by the three points in the section below. Set A and Set B then became the points projected from one or the other side, respectively, after which the modified Chamfer index was calculated for the two sets using the createTree() function of the SearchTrees package. The statistics used to calculate the asymmetricity between both sides were the difference in points between the two hemispheres, the difference in points/ total cilia points, the median and the mean modified Chamfer distance. In the end, they were visualized and clustered using a PCA to see trends (see code).

In the case of Figure 4E, instead of calculating the asymmetry statistics after getting the equation of the plane, we then used the Rodrigues’ rotation formula to rotate the normal (and thus the plane) with the fixed intersection being the center of the bot. Rotations of 45, 90 and 135 degrees were used yielding 4 axes (in the form of plane equations) including the movement axis. To eliminate the z‐axis, we used a PCA to get the rotation matrix to convert the projected cilia points from 3D to 2D and calculated the Chamfer distance using the formula described at https://github.com/UM‐ARM‐Lab/Chamfer‐Distance‐API , except that we did not square point distances. This statistic was calculated along for the cilia of all linear and circulars and put into a paired Wilcoxon rank‐sum test with an alternative hypothesis of “greater” and “less” for circulars and linear respectively to see if the movement axis was “more asymmetrical” or “less asymmetrical” respectively. For the body we did the same procedure, with the exception that our statistic now involved finding the distance of the body points from the center of the bot (once again segregated into two hemispheres with the dot product). Then, we used the KS test to calculate a D‐statistic which had greater values the more dissimilar the two distance distributions for both hemispheres were. Just like the cilia we then used a paired Wilcoxon rank‐sum test with an alternative hypothesis of “greater” and “less” for circulars and linear respectively to see if the movement axis was “more asymmetrical” or “less asymmetrical” respectively.

### Neuronal Culture

We followed a previously established protocol for creating the neuronal cultures^[^ ^28^ ^]^ which is summarized as follows. A 150 cm dish was first coated with 0.1% gelatin for 20 min and then aspirated off before seeding mouse embryonic fibroblasts (ATCC #SCRC‐1008) in mouse embryonic fibroblast (MEF) growth media (89% DMEM GlutaMAX, 10% Fetal Bovine Serum (FBS), and 1% Anti‐anti). Once the MEFs were confluent, they were inactivated by adding 20 mL of MEF growth media containing 500 µL of 10 µg mL^−1^ mitomycin C (Sigma #M4287) and incubating for 2 and 3 h at 37 °C. After incubation, the MEF growth media + mitomycin C media was replaced with hiNSCs at a density of 1/10 of a confluent target vessel in 25 mL of hiNSC growth media (77.6% Knockout DMEM, 20.20% KnockOut Serum Replacement (KOSR), 1% GlutaMAX, 1% Anti‐anti, 0.18% 2‐mercaptoethanol with 0.1% of 20 ng mL^−1^ bFGF). The day after seeding the hiNSCs required a media change where all the old media was aspirated off, and 25 mL of fresh hiNSCs growth media was added. Media changes were performed every other day until the hiNSCs were 80%–85% confluent. 3 h before performing the differentiation, the destination vessels were first coated with .1 mg mL^−1^ poly‐d‐lysine (PDL) (enough to coat the bottom of the wells) for 1 h at room temp, and then the PDL was aspirated before adding in 10 ug mL^−1^ laminin in DPBS (enough to coat the bottom) for 2 h at 37 °C. In the differentiation, the hiNSCs first went through one DPBS wash before adding TrypLE Select for 3–5 min to detach the cells from the plate. The cells were then collected and spun down for 3 min at 500 g then resuspended in neurobasal differentiation media (96% Neurobasal Media, 2% B‐27 supplement, 1% GlutaMAX, 1% Anti‐anti). The hiNSCs were seeded at a concentration of 100 000 cells cm^−2^. Once the hiNSCs were in differentiation, there was a media change the day preceding their differentiation and then every other day from there.

### Traversal Video Tracking and Analysis

To better explain the relationship between bot trajectory and movement in certain environments, we tried to relate the scratch edge to the actual movement of the bot. The steps taken before analysis involved i) creating a background image prototype from the.czi recording, ii) verifying the quality of the background image and saving as .png file, iii) tracking the bot, iv) extracting the coordinates of the scratch, and v) checking whether tracking was correctly carried out by generating a video with a beacon on the bot.

This procedure was carried out on 30+ files and yielded 20 usable datasets, which were whittled down to 17 after a manual check of tracking quality and excluding videos where bots never touched the scratch wall. Using the coordinates of the scratch walls and the tracking of the bot, we used the Rbioformats (version 0.0.74)^[^ ^44^ ^]^ and Revision (version 0.6.2)^[^ ^45^ ^]^ package tools to test 1) whether bots were more in contact with the scratch when they have a higher rotational tendency and 2) when moving on tissue, whether faster bots tend to cover more area, i.e., explore better. The lm() function was used to model the data after calculation of proportion of bot on tissue, instantaneous angular velocity and linear speed as variables. Before each model was approved, diagnostics were run on the model using the DHARMa package which included analysis of the residuals.

Afterwards, in order to take a better look at the nuances of interactions between bots and scratches, we decided to limit the data and remove any videos that had bots with very low rotational tendency (<0.33) since they were not stable enough in their rotational behavior. Bots with very high rotational tendency (>0.7) were removed since they were prone to skidding instead of interacting with the scratch walls. Finally, bots whose tracking videos were not optimal i.e., they frequently went backward or circularly in the scratch were removed since they did not have consistent forward movement that could be correlated with the scratch wall. After all these removals, our dataset ended up with 13 examples of scratch‐bot interactions which could be effectively analyzed. The Gyration was simply the Rotational Tendency values renamed. The Scratch‐Trajectory Similarity metric was calculated as the larger absolute value of the correlation between the heading angle of the trajectory and the heading angles of the scratch from the surface perpendicular to the bot. These correlation values were then modelled using the lm() function with an expectation of a quadratic relationship for Gyration. The specifics can be seen in the attached code.

### Traversal Video Processing

Traversal videos of bots moving along a scratch within a neuron plate were processed via Adobe Illustrator to see if the bot faithfully followed the edge of the scratch. The first method of processing aligned the center of the scratch at a horizontal line parallel to the bottom of the screen and placed a point on the center of the bot at each frame of the video as well as straight above and below this point on the edges of the scratch. Lines were made to connect each respective type of point for both of the edges of the scratch and the position of the bot. The output of this for further analysis was a set of coordinates of the end of each line derived from rendering these series of lines as an vector file and exported as text.

### Neuronal Tissue Density Analysis

To investigate whether these “bridges” were actually akin to neurons, we decided to analyze the pixel densities of various areas on and surrounding the bridge. In order to prevent confusions regarding this process with regard to intensity of color, we binarized the image on ImageJ. If the automatic thresholding did not visually appear similar to the raw image, we adjusted the threshold manually. We ended up with six areas of interest: the neurons above the bridge, below the bridge, to the left but adjacent to the left but far, and to the right, both adjacent and far. These areas were defined relative to a FIJI ROI box on the neuronal bridge which tried to encompass the width of the bridge and the height close to the narrowest point of the scratch channel that we would interact with. A line of one bridge length or lower if the image size required smaller lines to fit the boxes was used in the vertical and horizontal directions (called hereafter as “bridge length”). The above and below bridge measurements were taken by placing the bounding box one vertical bridge length from the box on the neuronal ridge. The adjacent areas on both sides were defined as 1 horizontal bridge length away from the bridge in the scratch. The far areas were 1 bridge length beyond the adjacent areas. The far and adjacent boxes were (vertically) adjusted so they overlaid the scratch as much as possible (Figure S9, Supporting Information). Finally, we used Analyze>Histogram in FIJI to get the size of the box (which was constant) and the number of pixels of scratch tissue (in black) and calculated the proportion. We then used an unpaired two sample T‐test with unequal standard deviation to calculate the significance of the difference, if any.

### Statistical Analysis

For all analyses in the paper, the p‐value to symbol correspondence was that a range of 0 to 0.0001 corresponded to ****, 0.0001 to 0.001 corresponded to ***, 0.001 to 0.01 corresponded to **, 0.01 to 0.05 corresponded to * and 0.05 to 1 corresponded to ns. Additionally, all significance tests were evaluated at an alpha value of 0.05. Unless otherwise specified, the alternative hypothesis was always two‐sided for t‐tests. For all statistical analyses listed below we used the Rstudio computational/ statistical software.

For Figure 2, we analyzed tracks from 197 bots for 5 h, collected across 47 timelapse videos (each video featuring 4–5 bots). In the pre‐processing step, we omitted data that is within one bot length (≈100 um) from the edge of the vessel to prevent edge effect as a confounding factor, which yielded a final of 42 235 individual 30 s periods. After cross‐entropy clustering these periods, we used a t‐test to analyze cluster‐specific differences in active periods (with cluster 1 having 6004 periods, cluster 2 with 6700, cluster 3 with 3436 and cluster 4 with 2384), which were further analyzed as shown on the figure. There were 23 711 inactive periods that were excluded from this downstream analysis.

For Figure 3, the pre‐processing step involved binarizing cilia versus body masses of 350 bots (each represented in 3D via confocal Z‐stack images) through CiliaQ^[^ ^38^ ^]^ as described in the methods above. The data obtained from these 350 bots were further clustered into 3 groups with sizes of 125, 24 and 201 for clusters 1,2 and 3 respectively. To check which of the 8 variables that were used to compute the PCA were significant for each cluster, we ran a two‐sided, two‐sample t‐test on all pairs of clusters, for all 8 variables.

For Figure 4, we used the same full set of data for 350 bots as Figure 3, which were still clustered into 3 groups with sizes of 125, 24 and 201 for clusters 1,2 and 3 respectively. Of these 350, it had specific information on type of movement for 28 bots (“displacers” – circulars and linears). Thus, of these 350, we then focused on 28 bots, 15 circular and 13 straight. These were analyzed for various metrics of asymmetry (difference between the two hemispheres in number of cilia, or chamfer distance of the hemispheres of cilia etc.) along the movement axis and the axis that was 90 degrees offset from the movement axis as described in methods above. We then measured the change in the chamfer distance asymmetry statistic from the 90‐degree offset to the movement axis (asymmetry difference = asymmetry ≈90 degree offset – asymmetry around the movement axis) for circulars (n = 15) and linears (n = 13) with a two‐sided one sample t‐test for each with a significant result for circulars (p = 0.0482) but not linears (p = 0.1116).

For Figure 5 during preprocessing, we excluded videos where the bot never touched the scratch wall as described in the methods above. A t‐test for the slope was run on the relationship between bots’ rotational tendency and proportion of bot on tissue which yielded a significant (p = 0.017, slope 1.15, n = 17) result. Similarly, when we compared the relationship between bot linear speed and the proportion of bot on tissue using a t‐test again, we received a significant result (p = 0.031, slope 0.0082, n = 17). For a subset of these 17 bots (dataset constrained to non‐stalling bots with rotational tendencies between 0.33 and 0.7 and viable tracking videos as described in methods above), we initially tried to fit a linear model of the relationship between bot rotational tendency and scratch‐trajectory similarity metric. The residuals of this analysis were not centered around a mean of 0 but rather followed a visibly quadratic trend (Figure S9, Supporting Information). This suggested a quadratic model would be a better fit for the relationship between the two. We ran the t‐test for the significance of this quadratic relationship that was significant (p = 0.006, n = 13).

For Figure 6, we ran a two‐sample t‐test pairwise with each category to characterize the pixel density of these various areas (gap closure site, native tissue, sites adjacent and distal to the gap closure sites) for all bridges where the connectivity to both sides of the scratch was maintained throughout the 3‐day experiment, which was 50% of the total N = 10. Difference between gap closure site and native tissue is insignificant (p = 0.37), while the difference between the gap closure site and both adjacent and distal scratch sites are significant (w/ p = 0.006 and p = 0.005, respectively).

## Conflict of Interest

This work is partially funded by a sponsored research agreement between Tufts University and a company called Astonishing Labs; co‐author Levin is a scientific co‐founder of Astonishing Labs.

## Author Contributions

P.S., and B.G.C. contributed equally to this work. G.G., and M.L. performed experimental design and data interpretation. G.G., P.S., B.C., H.L., B.S., S.G. performed data acquisition and analysis. All co‐authors Wrote the manuscript. M.L. performed funding acquisition.

## Supporting information

Supporting Information

Supplemental Video 1

Supplemental Video 2

Supplemental Video 3

Supplemental Video 4

Supplemental Video 5

Supplemental Video 6

Supplemental Video 7

## Data Availability

The data that support the findings of this study are available from the corresponding author upon reasonable request.
