# Supplementary material for: Motile Living Biobots Self‐Construct from Adult Human Somatic Progenitor Seed Cells
Source: Adv Sci (Weinh). 2023 Nov 30;11(4):2303575. doi: 10.1002/advs.202303575 (PMC10811512; doi:10.1002/advs.202303575)
Supplement: Supplementary file 1 — Supporting Information [file ADVS-11-2303575-s008.pdf]

ADVANCED SCIENCE
Open Access

# Supporting Information

for *Adv. Sci.*, DOI 10.1002/advs.202303575

Motile Living Biobots Self-Construct from Adult Human Somatic Progenitor Seed Cells

*Gizem Gumuskaya*, Pranjal Srivastava, Ben G. Cooper, Hannah Lesser, Ben Semegran, Simon Garnier and Michael Levin**

# Supplemental Figures

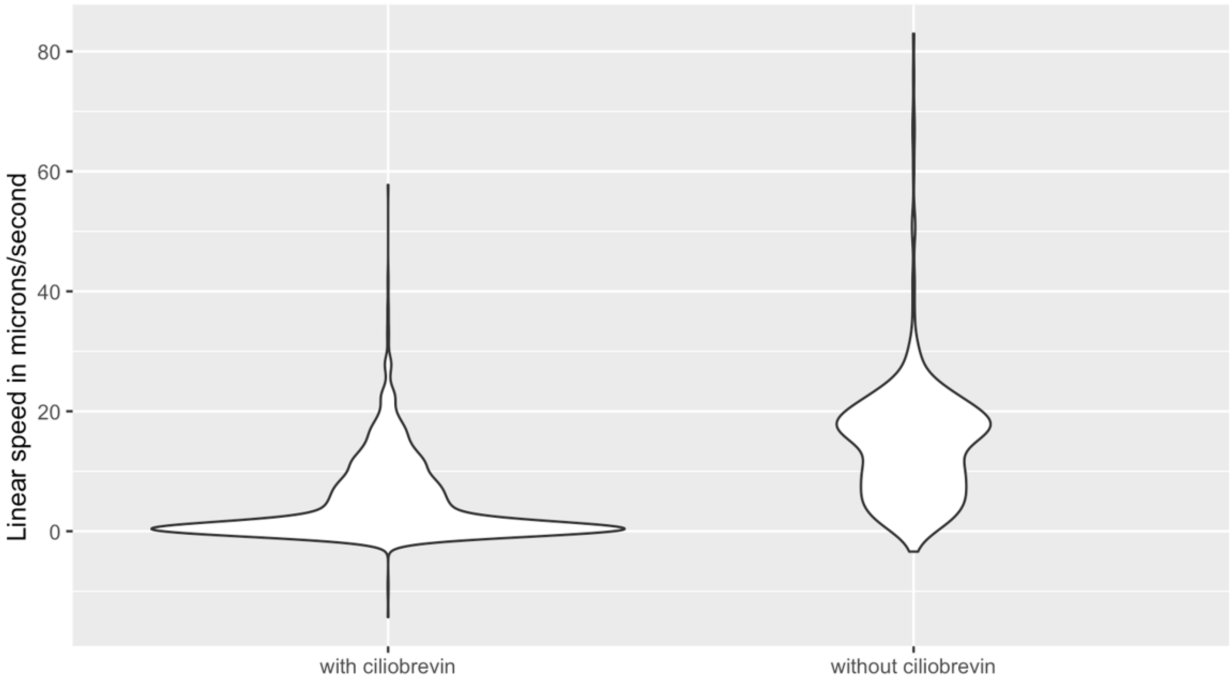


**Figure S1** Characterization of motility in terms of linear speed right after and prior to administration of ciliobrevin, a cilia-blocking agent. Significant drop in speed ($p<0.0001$, using T-test) is observed.

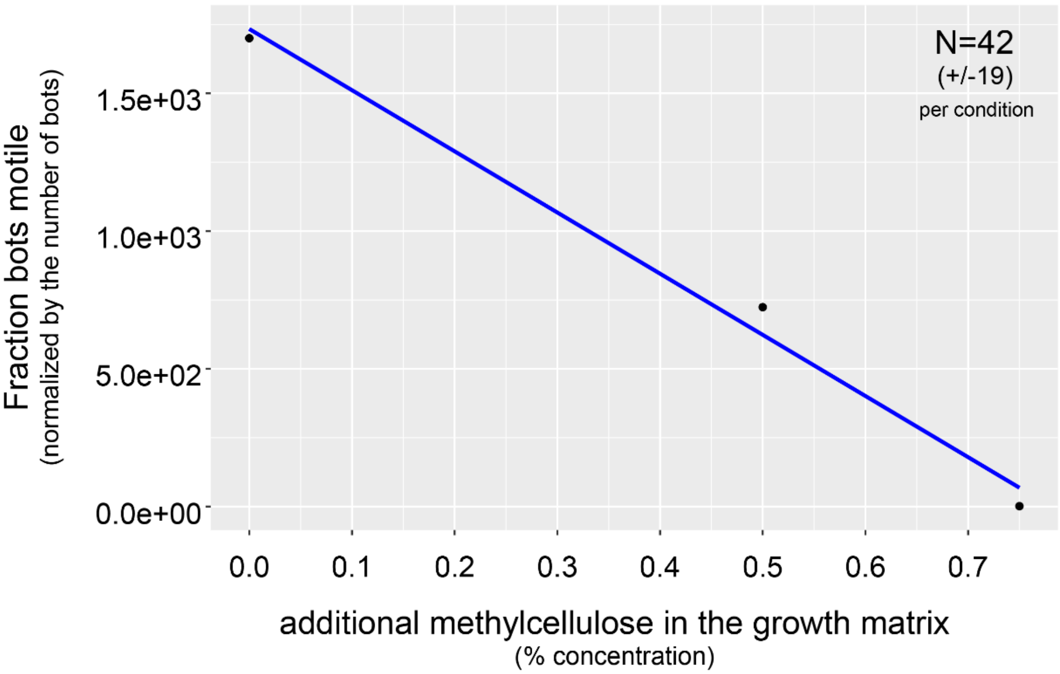

**Figure S2** Fraction of bots that show motility decreases as the matrix viscosity increases (as mediated by addition of methyl cellulose into the Matrigel). Resultant linear regression of matrix viscosity (0 vs 0.5 vs. 0.75%) as compared to the # of motile bots (weighed by the total number of bots) revealed a linear decreasing trend with a significant negative slope (p = 0.044).

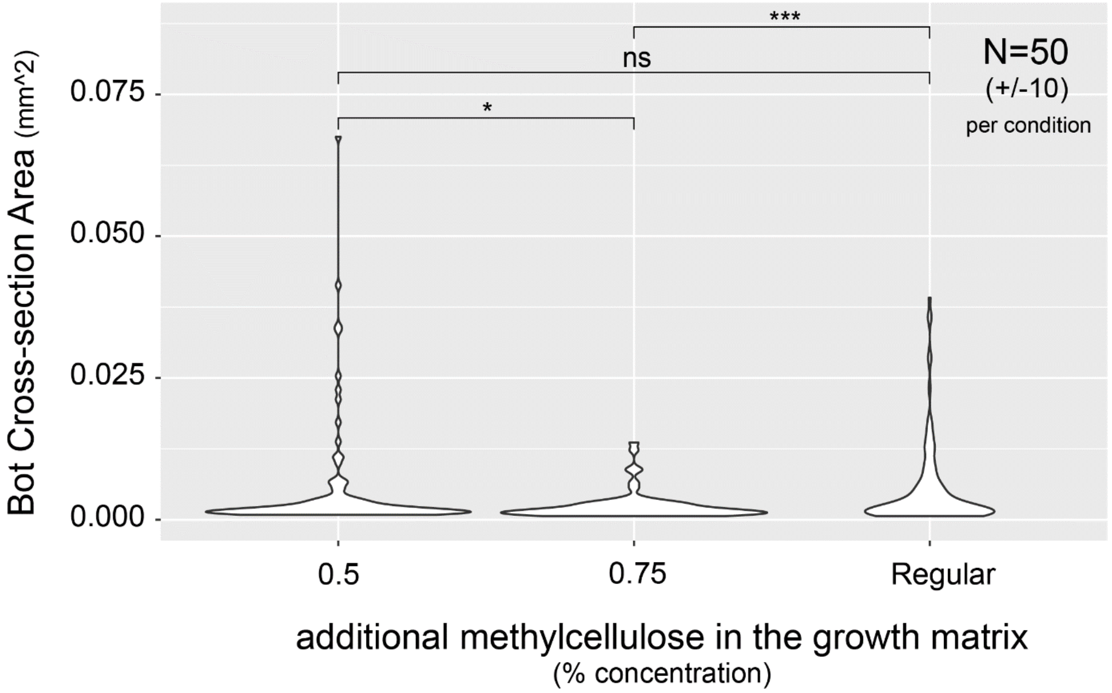


**Figure S3** Matrix viscosity (as modulated by addition of methyl cellulose into the Matrigel) influences the size of Anthrobots. While reducing the medium density by a factor of two did not significantly affect the area of the bots produced (p = 0.84), the density of 75% of baseline medium resulted in significantly smaller bot sizes when compared with bots from both 0.5 and baseline densities (p = 0.016 and p = 0.0003 respectively).

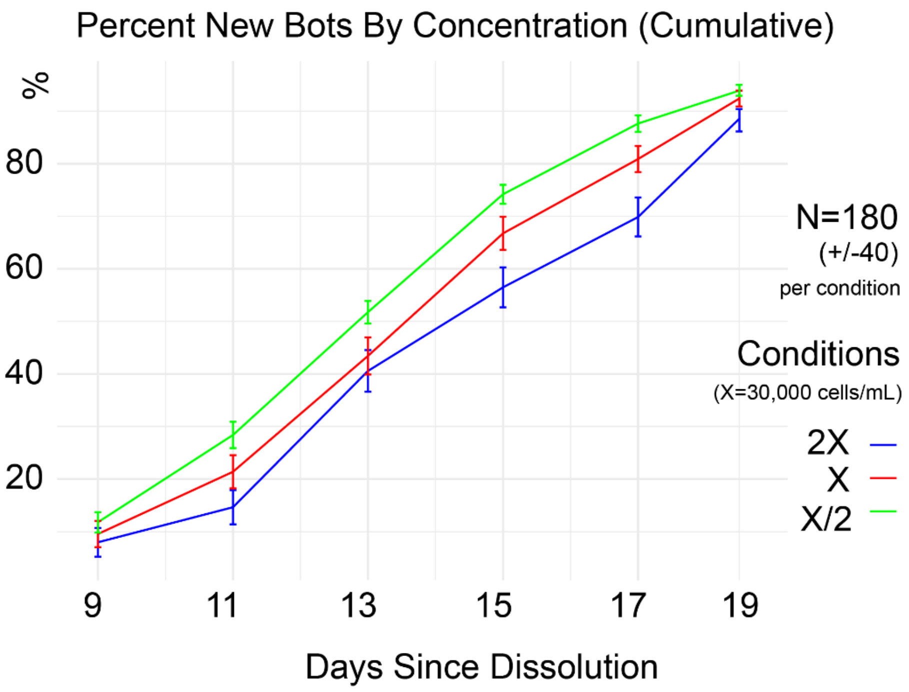


**Figure S4** Initial seeding density of individual cells is a key variable that influences the fraction of spheroids reaching motility at a given timepoint. We allowed bots to form as in the described protocol at a baseline concentration of 30,000cells/mL (X), as well as varied this number to half (X/2) and double (2X) and counted the number of spheroids becoming motile each day. We saw that the 0.5x concentration formed significantly more bots than the 2x concentration on Day 11-Day 20 with p values ranging from $<0.0001$ to 0.0206 for individual time points measured (Day 11,13,15,17,20 respectively). On Days 15 and 17, the Regular concentration formed significantly more bots than 2x concentration with $p = 0.0434$ and $p = 0.0184$ respectively. The 0.5x concentration formed significantly more bots than the baseline concentration on Day 17 with $p = 0.0323$.

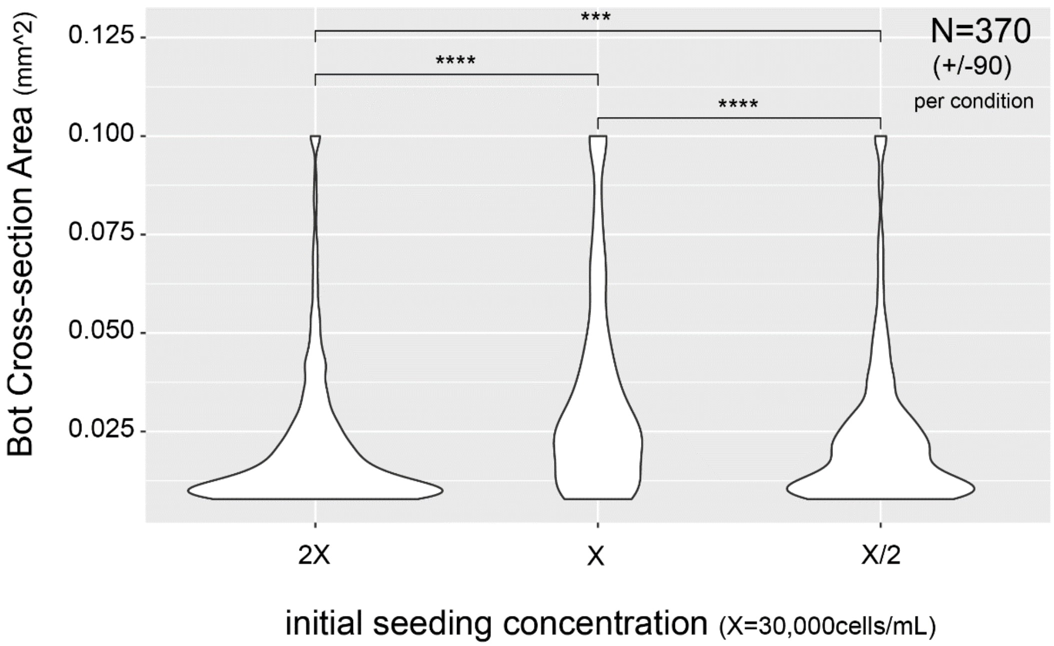


**Figure S5** Initial seeding density of individual cells as a key variable that may influence the size of Anthrobots. We allowed bots to form as in the described protocol at a concentration of 30,000cells/mL (X), as well as varied this number to half (X/2) and double (2X) and measured the average size immediately upon dissolution from matrix. The size of the formed bots was drastically different, with the average areas being 2.04*10^-4, 3.35*10^-4 and 2.37*10^-4 cm^2 for 2x, Regular and 0.5x concentrations respectively. These averages were all statistically distinct from each other, with $p < 2.2e-16$ for 2x vs. Regular, $p = 0.0002469$ for 2x vs. 0.5x and $p = 4.494*10^{-13}$ for Regular vs. 0.5x. These results suggest that our experimental concentration does affect size, and extreme concentrations lead to smaller bots in general.

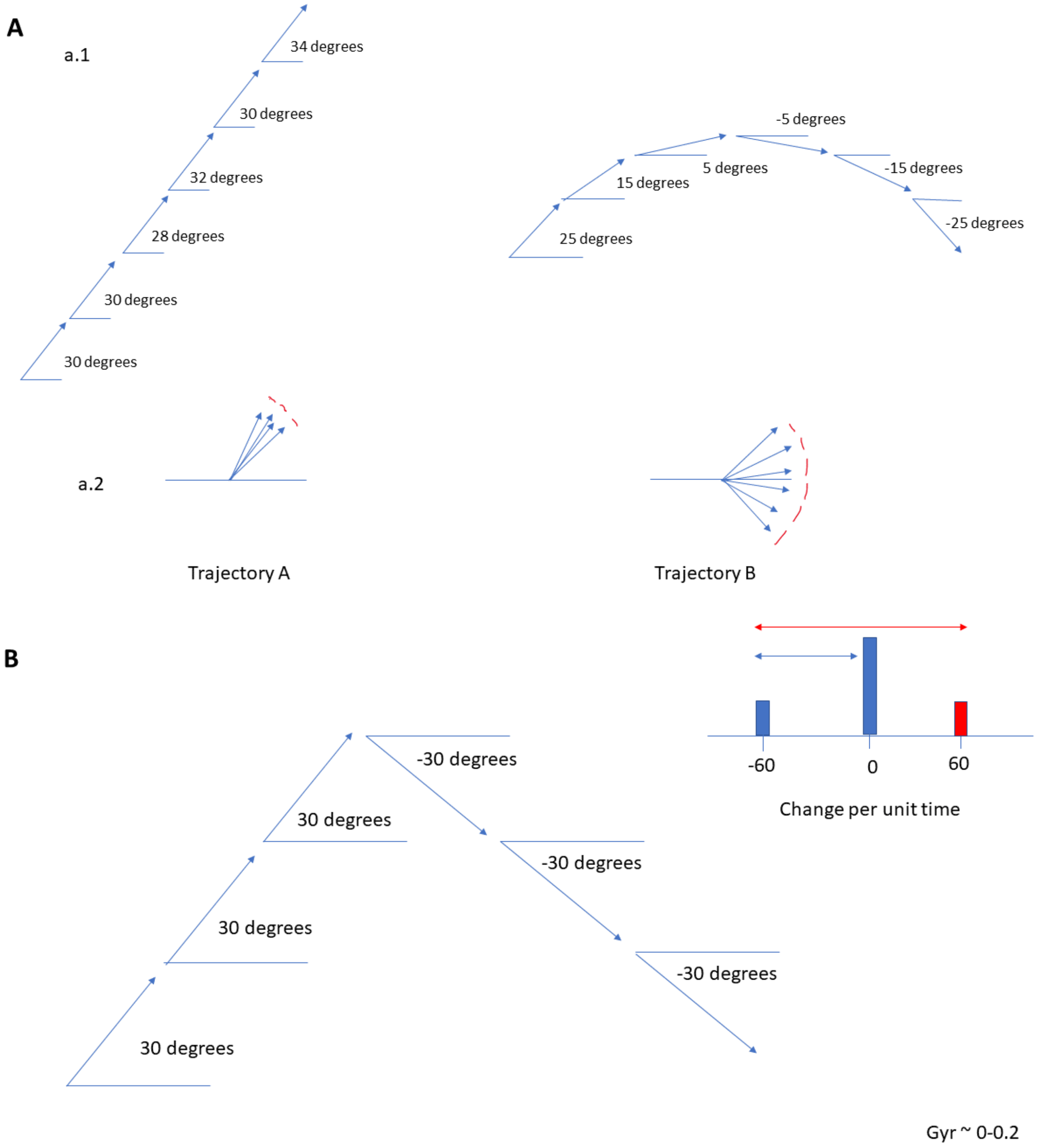

A
a.1
34 degrees
30 degrees
32 degrees
28 degrees
30 degrees
30 degrees
-5 degrees
5 degrees
15 degrees
-15 degrees
25 degrees
-25 degrees
a.2
Trajectory A
Trajectory B
B
-60
0
60
Change per unit time
30 degrees
-30 degrees
30 degrees
-30 degrees
30 degrees
-30 degrees
Gyr ~ 0-0.2

C

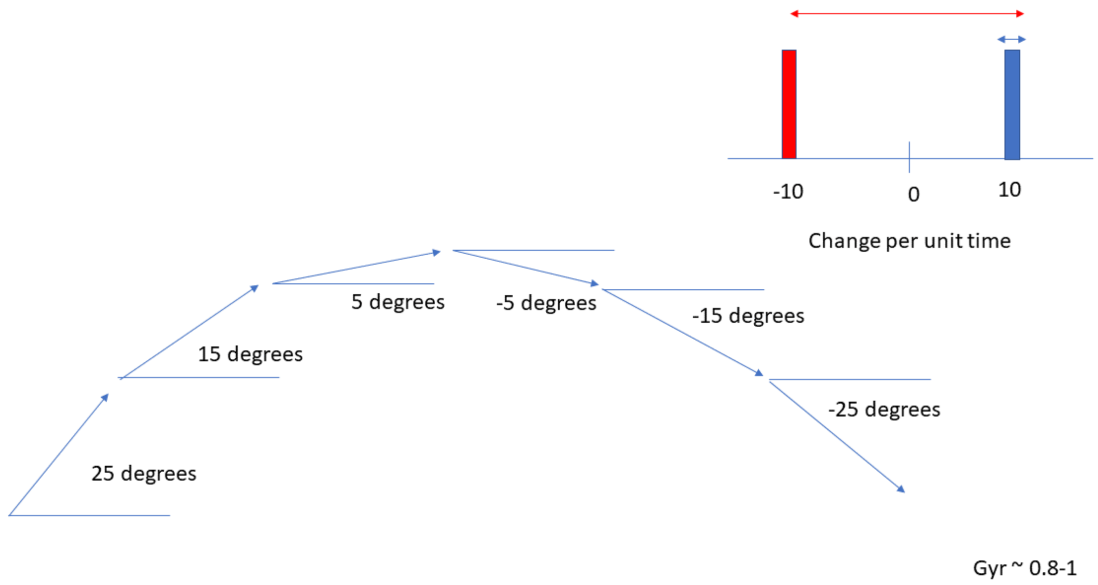


D

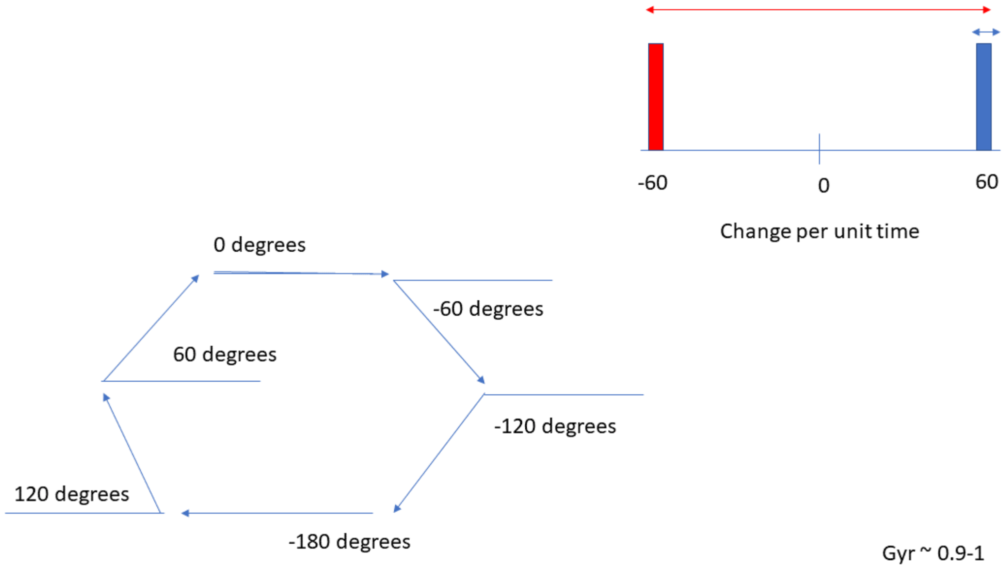


**Figure S6** Sample trajectories that reference a relative x-axis to find heading angle. (A) Visual representation of straightness index, which calculates spread of headings as a whole without taking into account temporal dynamics. (B, C, D) Visual representation of gyration index, which includes the temporal aspect and calculates spread of change in headings relative to their magnitude. Graphically, it is represented as the ratio of the blue length (original circular variance) to the red length (circular variance of the original angular speeds and their additive inverse) on the histograms.

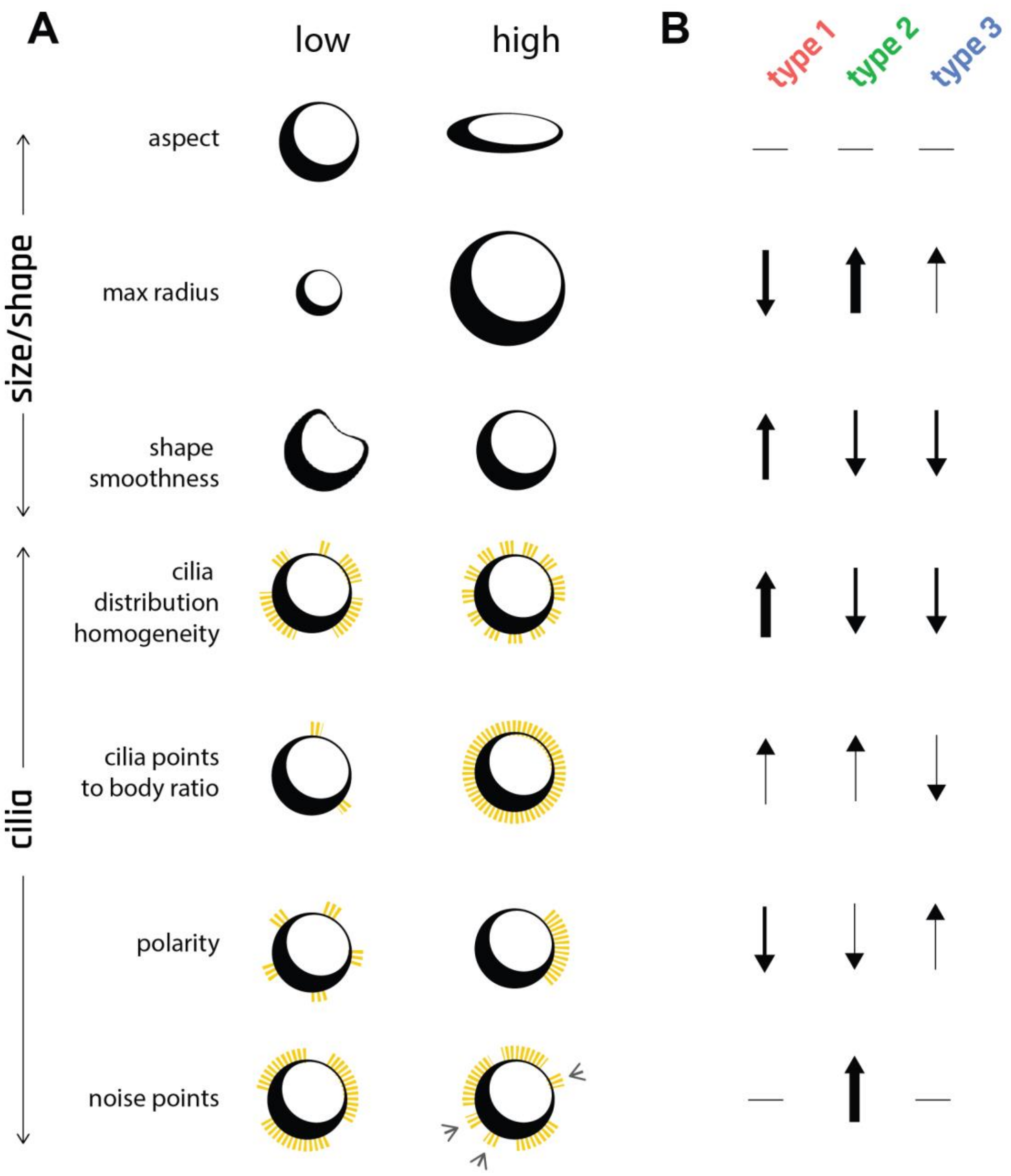


**Figure S7** Eight morphological indices were used to characterize Anthrobot morphotypes. (A) Visual summary of morphological indices at their extreme points (low, high). (B) Graphic summary of boxplots on Figure 3D, describing 3 different morphotypes. Arrow thicknesses are correlated with the # of standard deviations between a given morphotype's mean vs the overall population mean for a particular morphological index.

45 Degrees

<table>
<tr><td></td><td>Circular</td><td>Linear</td></tr>
<tr><td>Cilia</td><td>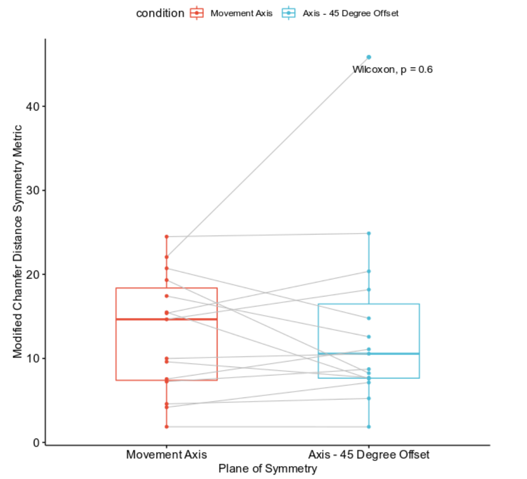
</td><td>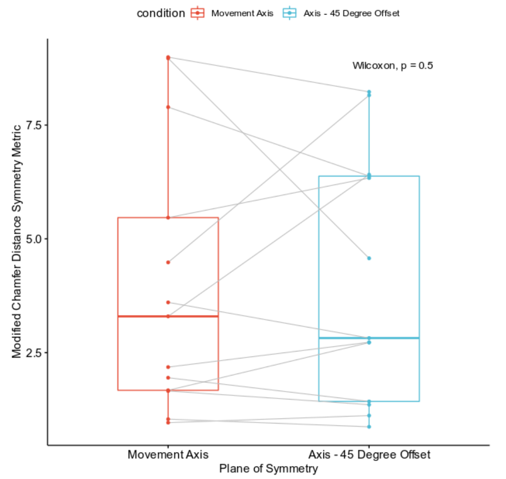
</td></tr>
<tr><td>Body</td><td>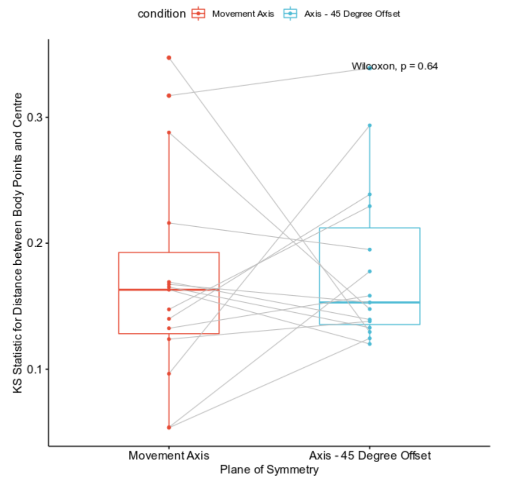
</td><td>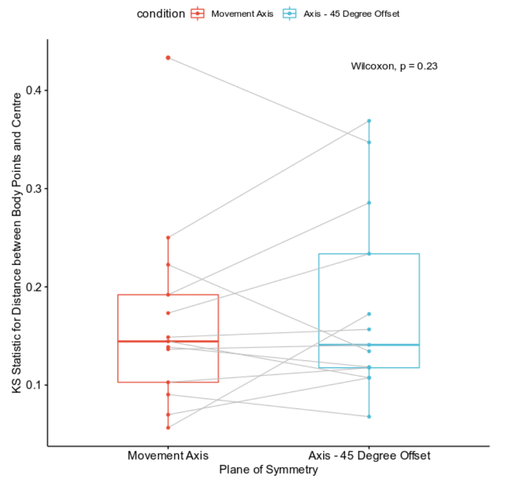
</td></tr>
</table>

90 Degrees

| | Circular | Linear |
|---|---|---|

<table>
<tr>
<td>Cilia</td>
<td>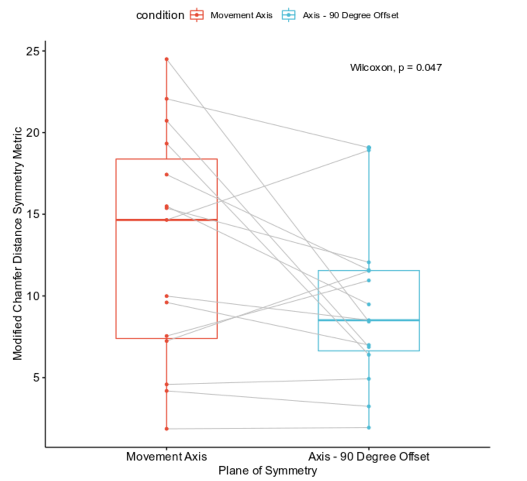
</td>
<td>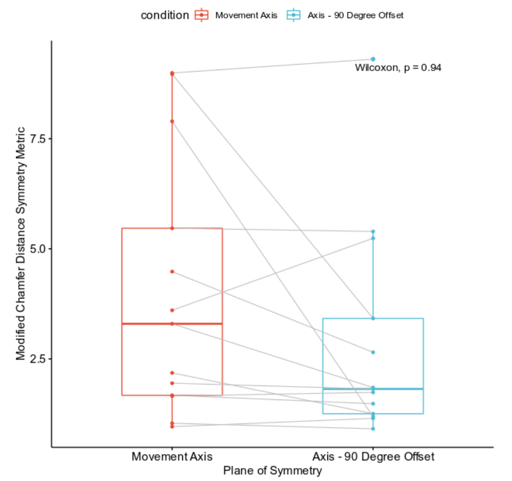
</td>
</tr>
<tr>
<td>Body</td>
<td>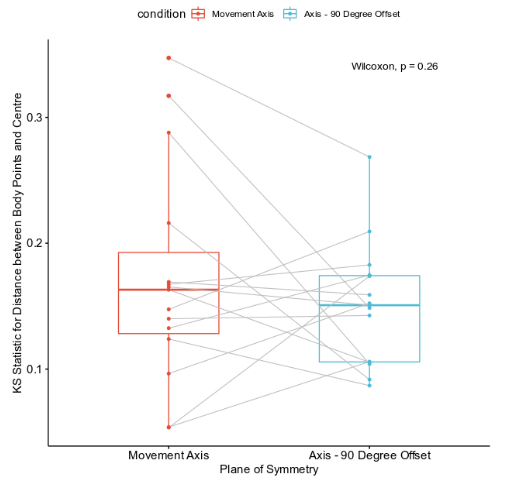
</td>
<td>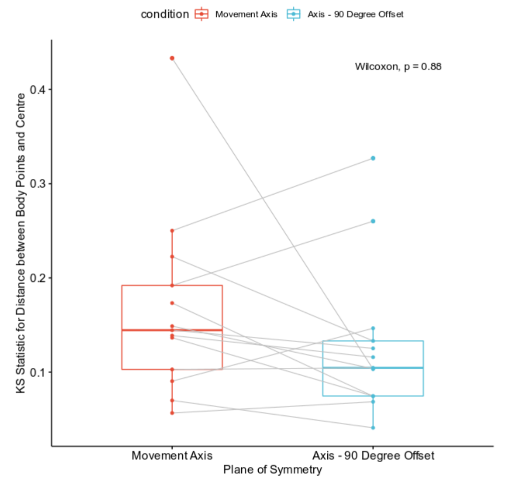
</td>
</tr>
</table>

135 Degrees

<table>
<tr><td></td><td>Circular</td><td>Linear</td></tr>
<tr><td>Cilia</td><td>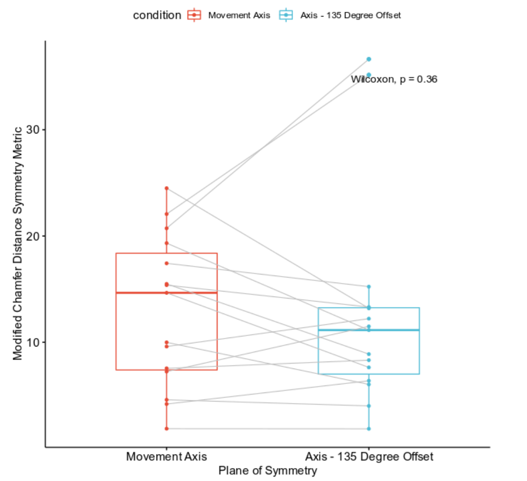
</td><td>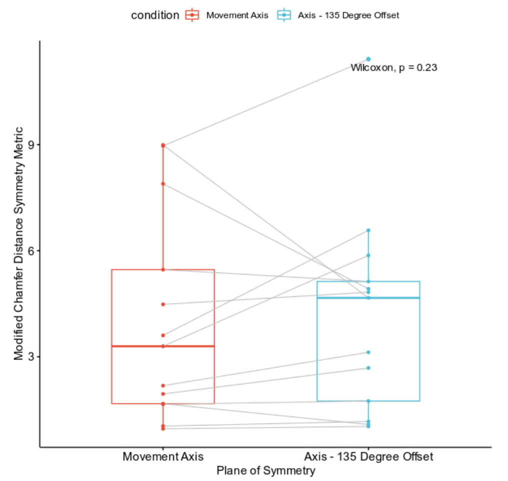
</td></tr>
<tr><td>Body</td><td>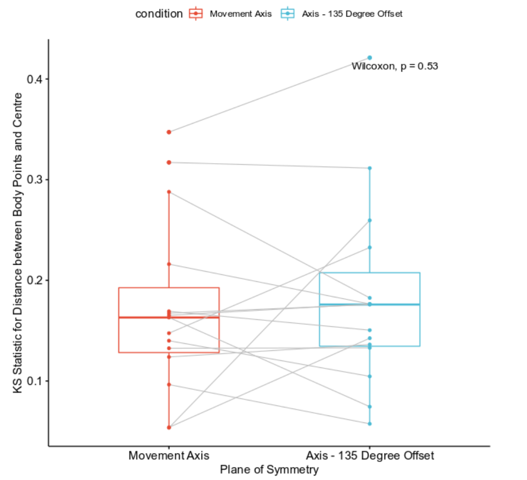
</td><td>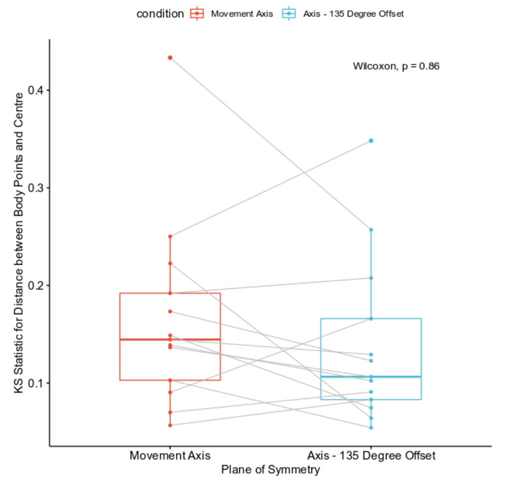
</td></tr>
</table>

**Figure S8** Difference in asymmetry of cilia distribution and body shape between linear and circular bots in respect to the movement axis and its 45, 90, and 135-degree offset axes.

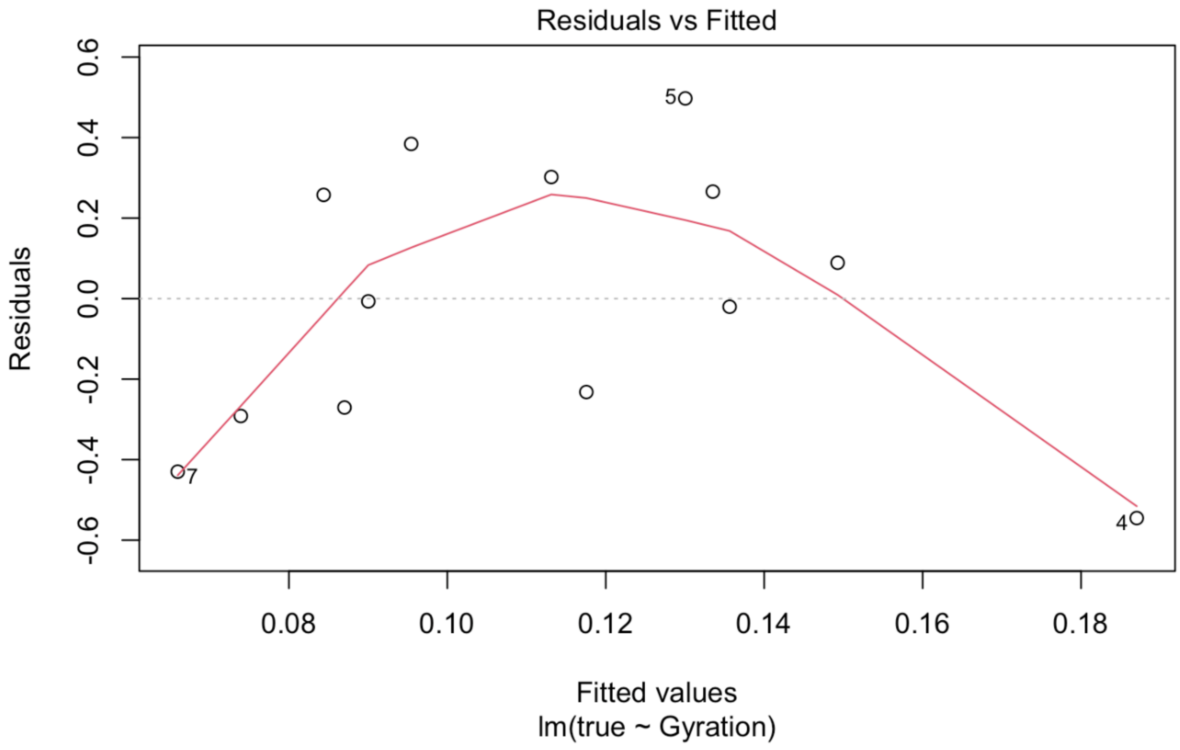

Residuals vs Fitted
Residuals
Fitted values
lm(true ~ Gyration)
0.6
0.4
0.2
0.0
-0.2
-0.4
-0.6
0.08
0.10
0.12
0.14
0.16
0.18
5
7
4

**Figure S9** To verify the relationship between the scar-trajectory similarity metric and the rotational tendency, we first used a linear model to measure the relationship between the two. The resulting residual vs. fitted values plot, a common diagnostic to check whether our model assumption (i.e., that the residuals have no trends and are centered around 0) are true. Here, the fitted values are the y values generated by evaluating our linear regression equation at the given x values (in our case, x is rotational tendency). The residuals are the actual y values (the actual scar-trajectory similarity metric) minus the predicted scar-trajectory similarity metric using the regression. When we plotted the residuals versus the fitted values for this model in this way, we observed a clear quadratic trend. Thus, this strongly suggested that a quadratic model is the most fitting to represent the relationship and that relationship was significant as shown on Figure 5 panel E of the manuscript. Furthermore, the fitness of the model, as measured by R^2, increased to 0.55 from 0.01 when we switched to quadratic from linear model, further suggesting quadratic model being an appropriate fit.

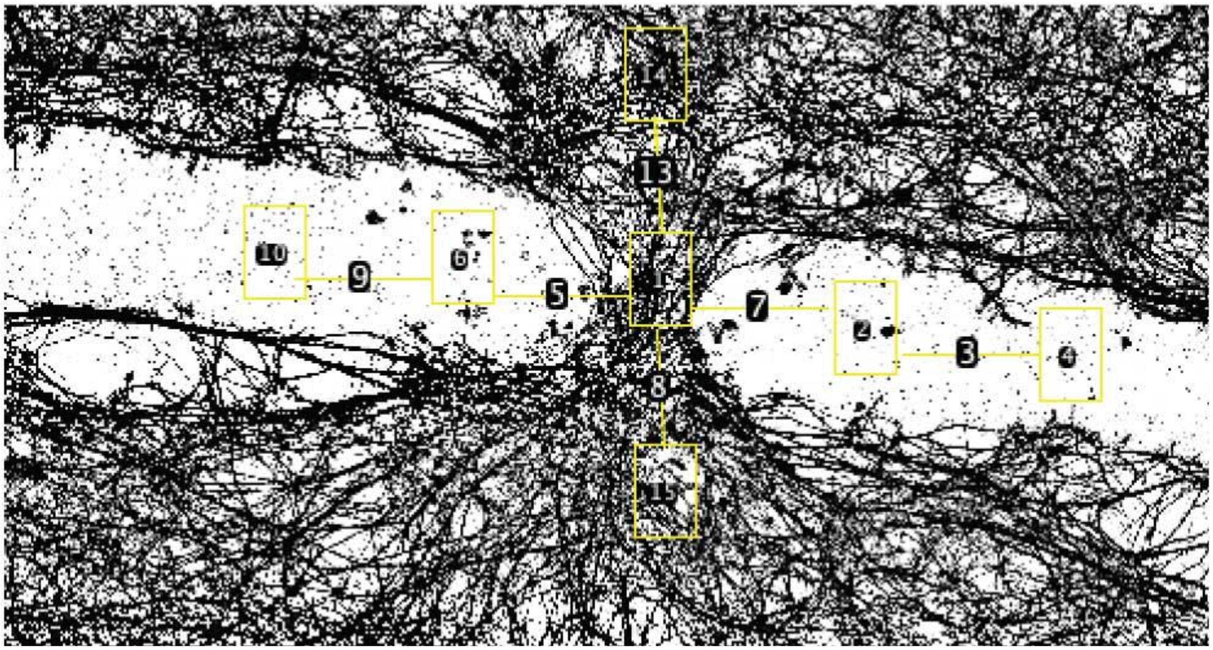


**Figure S10** Sample neuronal density sampling region. Each rectangle represents an area sampled and the lines are consistently the same length, the “bridge length.”

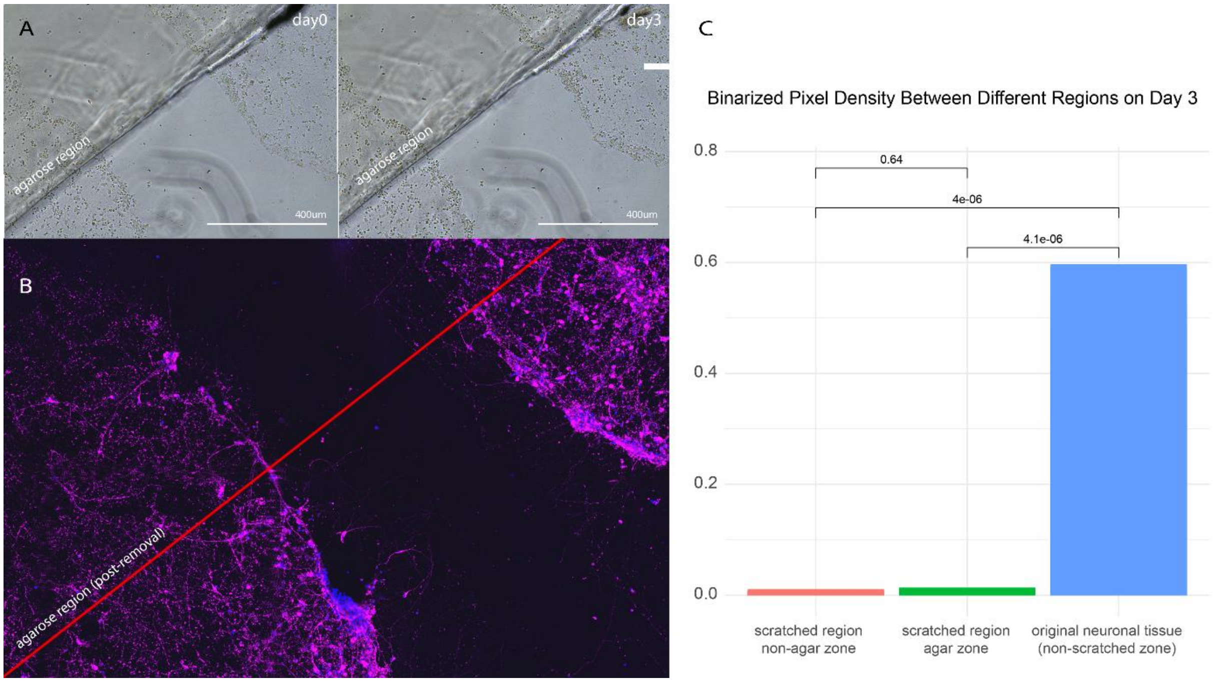


**Figure S11 (A)** Day 0 and 3 (i.e., day 4 upon scratching and agarose slab placement) images of a neuronal scratch with a piece of agarose slab (indicated with labels "agarose region") placed to test for the influence of passive mechanical loading (as opposed to Anthrobots) on gap closure, which induced no difference in neuronal density between the agarose loaded and non-loaded regions as identified by (B) TuJ1 neuronal staining upon fixation on day 3 as well as (C) image analysis on agarose loaded and non-loaded zones with N=10 replicates.

# Supplemental Videos

Have been directly uploaded to the submission portal. Please find below the legends and additional technical information for each video:

## Video Legends

**Supplemental Video 1 –** Close up of an Anthrobot featuring cilia as the locomotive appendage used for the propulsion strategy. Video collected via high-speed microscopy for 3 seconds and featured here at real time.

**Supplemental Video 2** - Anthrobots are self-constructing motile biobots, and therefore can be created in a high-throughput manner. Video collected at 2.5 seconds between each frame for 1 hour and featured here at 10FPS rate.

**Supplemental Video 3 -** Anthrobots exhibit a range of movement patterns. (Higher zoom, narrower arena.) Video collected at 2.5 seconds between each frame for 75 minutes and featured here at 40FPS rate.

**Supplemental Video 4 -** Anthrobots exhibit a range of movement patterns. (Lower zoom, broader arena.) Video collected at 2.5 seconds between each frame for 100 minutes and featured here at 40FPS rate.

**Supplemental Video 5 -** Sample linear mover Anthrobot. Video collected at 1 second between each frame for 4 minutes and featured here at 15FPS rate.

**Supplemental Video 6 -** Anthrobot with higher circular tendency traverse a neuronal scratch. Video collected at 2.5 seconds between each frame for 2 minutes and featured here at 5FPS rate.

**Supplemental Video 7 -** Anthrobot with lower circular tendency traverse a neuronal scratch. Video collected at 2.5 seconds between each frame for 2 minutes and featured here at 5FPS rate.
